# Absence of integrin α3β1 promotes the progression of HER2-driven breast cancer in vivo

**DOI:** 10.1186/s13058-019-1146-8

**Published:** 2019-05-17

**Authors:** Veronika Ramovs, Pablo Secades, Ji-Ying Song, Bram Thijssen, Maaike Kreft, Arnoud Sonnenberg

**Affiliations:** 1 Division of Cell Biology, The Netherlands Cancer Institute, Amsterdam, The Netherlands; 2 Department of Experimental Animal Pathology, The Netherlands Cancer Institute, Amsterdam, The Netherlands; 3 Oncode Institute and Department of Molecular Carcinogenesis, The Netherlands Cancer Institute, Amsterdam, The Netherlands

**Keywords:** Integrin α3β1, HER2, MMTV-cNeu, Metastasis, Breast cancer, Extracellular matrix, Collagen, Adhesion, Interstitial fluid flow

## Abstract

**Background:**

HER2-driven breast cancer is correlated with poor prognosis, especially during its later stages. Numerous studies have shown the importance of the integrin α3β1 during the initiation and progression of breast cancer; however, its role in this disease is complex and often opposite during different stages and in different types of tumors. In this study, we aim to elucidate the role of integrin α3β1 in a genetically engineered mouse model of HER2-driven mammary tumorigenesis.

**Methods:**

To investigate the role of α3β1 in HER2-driven tumorigenesis in vivo, we generated a HER2-driven MMTV-cNeu mouse model of mammary tumorigenesis with targeted deletion of Itga3 (Itga3 KO mice). We have further used several established triple-negative and HER2-overexpressing human mammary carcinoma cell lines and generated ITGA3-knockout cells to investigate the role of α3β1 in vitro. Invasion of cells was assessed using Matrigel- and Matrigel/collagen I-coated Transwell assays under static or interstitial fluid flow conditions. The role of α3β1 in initial adhesion to laminin and collagen was assessed using adhesion assays and immunofluorescence.

**Results:**

Tumor onset in mice was independent of the presence of α3β1. In contrast, the depletion of α3β1 reduced the survival of mice and increased tumor growth and vascularization. Furthermore, Itga3 KO mice were significantly more likely to develop lung metastases and had an increased metastatic burden compared to WT mice. In vitro, the deletion of ITGA3 caused a significant increase in the cellular invasion of HER2-overexpressing SKBR3, AU565, and BT474 cells, but not of triple-negative MDA-MB-231. This invasion suppressing function of α3β1 in HER2-driven cells depended on the composition of the extracellular matrix and the interstitial fluid flow.

**Conclusion:**

Downregulation of α3β1 in a HER2-driven mouse model and in HER2-overexpressing human mammary carcinoma cells promotes progression and invasiveness of tumors. The invasion-suppressive role of α3β1 was not observed in triple-negative mammary carcinoma cells, illustrating the tumor type-specific and complex function of α3β1 in breast cancer.

**Electronic supplementary material:**

The online version of this article (10.1186/s13058-019-1146-8) contains supplementary material, which is available to authorized users.

## Background

Gene amplification and/or overexpression of a member of the epidermal growth factor receptor family, epidermal growth factor receptor 2 (HER2), is observed in around 20% of invasive breast cancers [1]. Initial studies of transgenic mice expressing an activated Neu (i.e., the rat homolog of HER2) under the transcriptional control of the mouse mammary tumor virus (MMTV) promoter provided direct evidence that HER2 acts as a mammary oncogene [2, 3]. The phosphorylation of the intracellular tyrosine kinase domain of HER2 results in activation of PI3K/AKT and MAPK/ERK pathways, leading to increased cell proliferation and survival [4, 5]. Despite numerous studies and medical advances, HER2 overexpression and activation remain linked with poor prognosis due to their correlation with shorter disease-free intervals and an increased risk of metastasis [6, 7]. Therefore, there is a need to better understand HER2-driven breast cancer, especially at its late stages.

Integrins, a family of transmembrane glycoproteins consisting of 18 α- and 8 β-subunits that form 24 distinct heterodimeric receptors, play an important role in cancer progression. Integrins are primarily involved in cell-matrix adhesion and serve as mechanochemical transducers that generate biochemical signals [8, 9]. Over recent years, it has become clear that the function of integrins within both tumor cells and tumor environment is highly complex, which is reflected by the fact that they often play opposing roles in the initiation and progression of different tumor types [10]. This is especially prominent for integrin α3β1, a laminin-332- and laminin-511-binding integrin that is expressed mostly in the epithelia of the kidneys, lungs, intestine, skin, bladder, and stomach. Among others, α3β1 can be found in cell-cell contacts and focal adhesions (FAs), dynamic protein adhesion complexes that form mechanical links between the extracellular matrix and the actomyosin cytoskeleton [11]. The regulation of FAs and consequent reorganization of the associated actin cytoskeleton are important determinants for cell migration. The presence of α3β1 has been associated with the promotion and suppression of different stages and diverse types of tumors through its interactions with integrin-associated proteins (such as tetraspanin CD151), changes in cell adhesion and/or migration, or via the induction of α3β1-mediated signaling [12]. Independent studies looking for correlations between α3β1 and breast cancer in selections of human tumor samples reported all possible outcomes—lack of correlation [13], positive correlation with tumor progression and angiogenesis [14], and correlation between the downregulation of α3β1 and increased invasiveness, resulting in reduced survival [15–17]. This illustrates that there is a need to investigate its clinical significance in relation to the phenotypical and histological variants of specific types of tumors.

Integrin α3β1 was shown to be essential for the initiation, proliferation, and invasiveness of basal tumors that are adherent to the pre-existing or newly deposited laminin matrix [18, 19]. Furthermore, its role in promoting HER2-negative breast cancer in vivo and in vitro in human breast cancer cell line MDA-MB-231 has been demonstrated in several studies [19–21]. However, the role of α3β1 in HER2-driven mammary tumorigenesis has not been properly addressed yet. In this study, we investigated the impact of the α3β1 deletion in a mouse model of HER2-driven tumorigenesis in vivo and in human mammary carcinoma cell lines in vitro. With this, we aim to add to the understanding of the complex function of α3β1 as a breast cancer marker and therefore to its clinical potential.

## Methods

### Generation of mice

According to Mouse Genome Informatics (Jackson Laboratory), the names of MMTV-Cre; MMTV-cNeu (Itga3 WT) mice are Tg (MMTV-cre)4Mam; Tg (MMTV-Erbb2)NK1Mul. MMTV-Cre; MMTV-cNeu; *Itga3*^fl/fl^ (Itga3 KO) mice were generated by intercrossing MMTV-Cre; MMTV-cNeu mice with *Itga3* ^fl/fl^ (i.e., Itga3^tm1Son/tm1Son^ according to Mouse Genome Informatics). Mice were bred onto an FVB/N background.

### In vivo tumor analysis

Mice were examined twice a week for the presence of palpable mammary tumors, and tumor sizes were measured using calipers. Mice were sacrificed when the total tumor mass per mouse reached 4 cm^3^. At the end of the in vivo experiments, full necropsies were performed and tumor tissues and all the organs were collected, fixed in 10% neutral formalin, embedded into paraffin blocks, and subsequently sectioned and stained for hematoxylin and eosin and/or immunohistochemistry analysis. Alternatively, tumors were embedded in Tissue-Tek OCT (optimal cutting temperature) cryoprotectant for immunofluorescent analysis of cryo-preserved material.

### Cell culture

MDA-MB-231, SKBR3, AU565, BT-20, BT474, and Hs 578T carcinoma cell lines were obtained from the research group of L. F. A. Wessels and were authenticated by suppliers using short tandem repeat profiling [22]. MDA-MB-231, Hs 578T, SKBR3, and AU565 were cultured in RPMI, BT474 in Advanced DMEM F12, and BT-20 in MEM culture medium. All cell lines were cultured with 10% heat-inactivated FCS and antibiotics. Hs 578T were additionally cultured with 10 μg ml^−1^ insulin. All cells were cultured at 37 °C in a humidified, 5% CO_2_ atmosphere.

### Generation of integrin α3-deficient cells

The target sgRNA against ITGA3 (exon 1; 5′CGGTCGCGAGCTGCCCGCGA-3′) was cloned into pX330-U6-Chimeric_BB-CBh-hSpCas9 (a kind gift from Feng Zhang [23]; Addgene plasmid #42230). MDA-MB231, SKBR3, AU565, and BT474 cells were transiently transfected with this vector using Lipofectamine® 2000 (Invitrogen). Lipofectamine (20 μl ml^−1^) and vector solution (3 μg) in Opti-MEM were mixed and incubated for 20 min at room temperature. Cells were incubated with the transfection solution overnight. Integrin α3-deficient cells were selected by fluorescent-activated cell sorting.

### Immunohistochemistry

After deparaffinization of the samples and antigen retrieval, tumor and lung tissue sections were consecutively stained with primary antibodies (see Table 1) and biotin-conjugated secondary antibodies, followed by incubation with streptavidin/HRP (DakoCytomation; P0397) and detection and visualization with DAB tablets (Sigma; D-5905). Images were taken with PL APO objectives (× 10/0.25 NA, × 40/0.95 NA, and × 63/1.4 NA oil) on an Axiovert S100/AxioCam HR color system using AxioVision 4 software (Carl Zeiss MicroImaging) or with the Aperio ScanScope (Aperio, Vista, CA, USA), using ImageScope software version 12.0.0 (Aperio).

**Table 1:** List of primary antibodies used, including application, dilution, and source

Antigen	Name	Type	Application	Dilution	Source
β-catenin	610154	Mouse mAb	IF	1:100	BD Bioscience
Actin	MAB1501R	Mouse mAb	WB	1:1000	Chemicon
Akt	9272	Rabbit mAb	WB	1:1000	Cell Signaling
Caspase3 (cleaved Asp 175)	9661 L	Rabbit pAb	IHC	1:500	Cell Signaling
CD31	ab28364	Rabbit pAb	IHC	1:500	Abcam
Collagen I	A67P	Rabbit pAb	IF	1:40	Chemicon
E-cadherin	610182	Mouse mAb	IF	1:100	BD Bioscience
GAPDH	CB1001	Mouse mAb	WB	1:1000	Calbiochem
HER2	2165S	Rabbit mAb	WB FACS	1:1000 1:200	Cell signaling
Itga2	10G11	Mouse mAb	FACS	1:100	[24]
Itga3	J143	Mouse mAb	FACS Functional assay	1:100 10 μg ml^−1^	[25]
Itga3		Rabbit pAb	WB	1:2000	Homemade
Itga3	A3-X8	Mouse mAb	Functional assay	10 μg ml^−1^	Kind gift of C. Stipp [26]
Itga6	GoH3	Rat mAb	FACS	1:200	[27]
Itgb4	346-11A	Rat mAb	IF	1:100	BD Bioscience
Keratin 5	PRB-160P	Rabbit mAb	IF	1:100	Covance
Keratin 18	RGE53	Mouse mAb	IF	1:2	Progen
Ki67	PSX1028	Rabbit pAb	IHC	1:750	Monosan
Laminin-332	R14	Rabbit pAb	IF	1:400	Kind gift of M. Aumailey
NEU	sc-284	Rabbit pAb	IHC	1:800	Santa Cruz
pAkt (Ser473)	4060	Rabbit mAb	IHC	1:10000	Cell Signaling
pAkt (Ser473)	9271	Rabbit mAb	WB	1:500	Cell Signaling
p4E-BP1 (Thr37/47)	2855	Rabbit mAb	IHC	1:1600	Cell Signaling
pErk1/2(Thr202/Tyr204)	4370	Rabbit mAb	IHC	1:400	Cell Signaling
Plet1	33A10	Rat mAb	IF	1:100	[28]
Tubulin	B-5-1-2	Mouse mAb	WB	1:5000	Sigma
Vinculin	VIIF9	Mouse mAb	IF	1:5	Kind gift of M. Glukhova

### Immunofluorescence

Cryosections of tumors were prepared, fixed in ice-cold acetone, and blocked with 2% bovine serum albumin (BSA, Sigma) in PBS for 1 h at room temperature. Tumor samples were incubated with the indicated primary antibodies in 2% BSA in PBS for 60 min, washed in PBS three times, and further incubated with secondary antibodies diluted 1:200 for 60 min. All samples were counterstained with DAPI for 5 min at room temperature and mounted in Vectashield (Vector Laboratories H-1000). Samples were analyzed by Leica TCS SP5 confocal microscope with a × 20 (NA 1.4) objective and processed using ImageJ [29, 30]. SKBR3 cells were fixed with 2% paraformaldehyde for 10 min, permeabilized with 0.2% Triton-X-100 for 5 min, and blocked with PBS containing 2% BSA for 1 h at room temperature. Cells were further incubated with the primary antibodies (see Table 1) for 1 h at room temperature, washed three times with PBS, and incubated with the secondary antibodies for 1 h. For integrin α3 and α2 staining, additional incubation with biotin-conjugated antibody was performed after primary antibody staining, which was followed by incubation with fluorophore-conjugated streptavidin. Additionally, the nuclei were stained with DAPI, and filamentous actin was visualized using Alexa Fluor 488-conjugated phalloidin (Invitrogen). After three washing steps with PBS, the coverslips were mounted onto glass slides in Mowiol. Images were obtained using a Leica TCS SP5 confocal microscope with a × 63 (NA 1.4) oil objective and processed using ImageJ [29, 30]. Focal adhesion size and amount were calculated using the Analyze Particle function, after drawing a region of interest (ROI) at the cell periphery (based on actin staining). The total cluster area was divided by the total ROI area to define focal adhesion area per cell.

### Western blot

Protein lysates for western blot analysis of tumors were obtained from FFPE tumor tissue samples by using Qproteome FFPE Tissue Kit (Qiagen) following the instructions of the manufacturer. Protein lysates of carcinoma cells were obtained from subconfluent cell cultures, washed in cold PBS, and lysed in RIPA buffer (20 mM Tris-HCl (pH 7.5), 100 mM NaCl, 4 mM EDTA (pH 7.5), 1% Nonidet P-40, 0.1% SDS, 0.5% sodium deoxycholate) supplemented with 1.5 mM Na_3_VO_4_, 15 mM NaF (Cell Signaling) and protease inhibitor cocktail (Sigma). Lysates were cleared by centrifugation at 14.000×*g* for 20 min at 4 °C and eluted in sample buffer (50 mM Tris-HCl pH 6.8, 2% SDS, 10% glycerol, 12.5 mM EDTA, 0.02% bromophenol blue) containing a final concentration of 2% β-mercaptoethanol and denatured at 95 °C for 10 min. Proteins were separated by electrophoresis using Bolt Novex 4–12% gradient Bis-Tris gels (Invitrogen), transferred to Immobilon-P transfer membranes (Millipore Corp), and blocked for 1 h in 2% BSA in TBST buffer (10 mM Tris (pH 7.5), 150 mM NaCl, and 0.3% Tween-20). The blocked membranes were incubated overnight at 4 °C with primary antibodies (see Table 1) diluted 1:1000 in TBST containing 2% BSA, after which they were washed twice with TBST and twice with TBS buffer. Next, the membranes were incubated for 1 h hour at room temperature with horseradish peroxidase-conjugated goat anti-mouse IgG or goat anti-rabbit IgG (diluted 1:5000 in 2% BSA in TBST buffer). After washing, the bound antibodies were detected by enhanced chemiluminescence using or Clarity™ Western ECL Substrate (Bio-Rad) or Amersham ECL Western Blotting Detection Reagent (GE Healthcare) as described by the manufacturer. Signal intensities were quantified using ImageJ [29, 30].

### Flow cytometry

Cells were trypsinized, washed in PBS containing 2% FCS, and incubated for 1 h at 4 °C in primary antibody in PBS 2% FCS. Next, the cells were washed twice in PBS containing 2% FCS and incubated with PE-conjugated donkey anti-mouse (Biolegend #406421; 1:200 dilution) or donkey anti-rat (Biolegend # 406421; 1:200 dilution) antibody for 30 min at 4 °C. After subsequent washing steps, cells were analyzed on a Becton Dickinson FACS Calibur analyzer. For fluorescent-activated cell sorting, α3-negative cell population was obtained using a Becton Dickinson FACSAria IIu cell sorter.

### Invasion assay

Transwell inserts with 8.0 μm pore polycarbonate membrane (Corning, #3422) were coated with 150 μl of either Matrigel (Corning® Matrigel® Growth Factor Reduced Basement Membrane Matrix, 3.3 times diluted in serum-free medium) or the mixture of Matrigel (3.3 times diluted in serum-free medium) and freshly prepared collagen I solution (1.05 mg ml^−1^), containing 20,000 cells, and left incubating for 1 h at 37 °C. When used, 4 μg of function-blocking or control antibodies was added to the gel. Collagen I solution was prepared by mixing 10 times the concentrated PBS, 1 M NaOH, and collagen I (2.8 mg ml^−1^, Advanced Biomatrix #5005), after which the mixture was incubated at 4 °C for 1 h. For interstitial fluid flow conditions, Transwell inserts were inserted in 24-well plate, containing 280 μl of cell culture medium supplemented with 10% FCS. Next, 450 μl of serum-free medium was gently pipetted on top of the gel into the Transwell inserts. When used, function-blocking or control antibodies were added to the serum-free medium at the concentration 10 μg ml^−1^. For static conditions, Transwell inserts were placed in 24-well plate containing 650 μl of cell culture medium supplemented with 10% FCS, and 150 μl of serum-free medium was pipetted into the Transwell insert. Cells were left to migrate for 21 h, after which the gel was aspirated, and the upper side of the membranes cleaned with cotton swabs. The membranes were then fixed in ice-cold methanol for 10 min and washed with PBS. Invading cells were stained with DAPI for 5 min at room temperature, and the total membranes were imaged with Zeiss Axio Observer Z1-inverted microscope, using automated tile imaging setting on Zeiss ZEN software and × 10 objective. Images were stitched and processed with Zeiss ZEN software and further analyzed using ImageJ [29, 30]. Circular ROI was selected in the central part of the membrane (115 mm^2^), and cells were quantified by counting DAPI-stained nuclei, using the Analyze Particle function.

### Adhesion assay

For adhesion assays, 96-well plates were coated with 3.2 μg ml^−1^ collagen I (Advanced Biomatrix #5005) or laminin-332-rich matrix. Collagen I coating was done in PBS solution at 37 °C for 1 h. Laminin-332-rich matrix was obtained by growing RAC-11P cells [31] to complete confluence, after which the plates were washed with PBS and incubated with 20 mM EDTA in PBS overnight at 4 °C. The RAC-11P cells were then removed by pipetting and washing with PBS, and the coated plates were kept at 4 °C in PBS until they were used. Before use, the coated plates were washed once with PBS and blocked with 2% BSA in PBS for 1 h at 37 °C. Carcinoma cells were trypsinized and resuspended in a serum-free cell culture medium. The cells were seeded at a density of 1 × 10^5^ cells per well and incubated for 30 min at 37 °C. Nonadherent cells were washed away with PBS, and the adherent cells were fixed with 4% paraformaldehyde for 10 min at room temperature, washed twice with H_2_O, stained with crystal violet for 10 min at room temperature, and washed extensively with H_2_O. Dried and stained cells were resuspended in 2% SDS, after which absorbance was measured at 595 nm on a Tecan infinite 200Pro microplate reader using Tecan i-control software.

### Antibodies

Primary antibodies used are listed in Table 1. Secondary antibodies were: goat anti-rabbit Alexa Fluor 488, goat anti-mouse Alexa Fluor 568, goat anti-rat Texas FITC, goat anti-rat Alexa Fluor 647 (Invitrogen), biotin-goat anti-mouse IgG, Cy-5 streptavidin (Zymed), PE-conjugated donkey anti-mouse antibody (Biolegend #406421), PE-conjugated donkey anti-rat antibody (Biolegend # 406421), stabilized goat anti-mouse HRP-conjugated, and stabilized goat anti-rabbit HRP-conjugated (Pierce).

### Breast cancer cell expression data analysis

We used the RNA sequencing gene expression data from Jastrzebski et al. [22] and from the Cancer Cell Line Encyclopedia [32, 33]. The read counts were normalized for library size and log-transformed. HER2 and ER status were annotated according to ATTC and ExPASy Cellosaurus [34], which matched with the presence of ERBB2 gene amplification and the level of ESR1 expression in the respective datasets. Cell lines were classified as luminal, basal, or post-EMT based on the annotation provided in [35]. For the cell lines not annotated in that reference, we used the same criteria to classify them as luminal, basal, or post-EMT, that is, cell lines with high KRT5 expression were classified as basal, and cell lines with high expression of VIM were classified as post-EMT. Gene-level copy number estimates were obtained from segmented copy number profiles by taking the log copy number ratio of the segment containing the start of the gene.

### Statistical analysis

Statistical analysis was performed using GraphPad Prism (version 7.0c). The graphs represent the mean and error bars standard deviation (SD) or standard error of mean (SEM), as indicated per graph. Unpaired two-tailed *t* test was used for comparisons of experimental groups with a control group, one-way ANOVA was used to compare multiple groups across a single condition, and chi-square test was used for categorical data. The statistical test used per experiment and significant values shown are described in appropriate figure legends. Results with *P* value lower than 0.05 were considered significantly different from the null hypothesis.

## Results

### Integrin α3β1 is not needed for the onset of HER2-driven mammary tumorigenesis

To investigate the role of integrin α3β1 in HER2-mediated mammary tumorigenesis and tumor progression in vivo, we employed the widely used breast cancer mouse model, MMTV-cNeu, designed to promote development of mammary tumors as a result of overexpression of HER2/Neu oncogene under the transcriptional control of the mouse mammary tumor virus (MMTV) promoter [2]. Additionally, mice harbored the MMTV-Cre transgene in the presence (MMTV-Cre; MMTV-cNeu; *Itga3*^fl/fl^ mice, i.e., Itga3 KO mice) or absence of floxed *Itga3* alleles (MMTV-Cre; MMTV-cNeu mice, i.e., Itga3 WT mice).

**Fig. 1:**
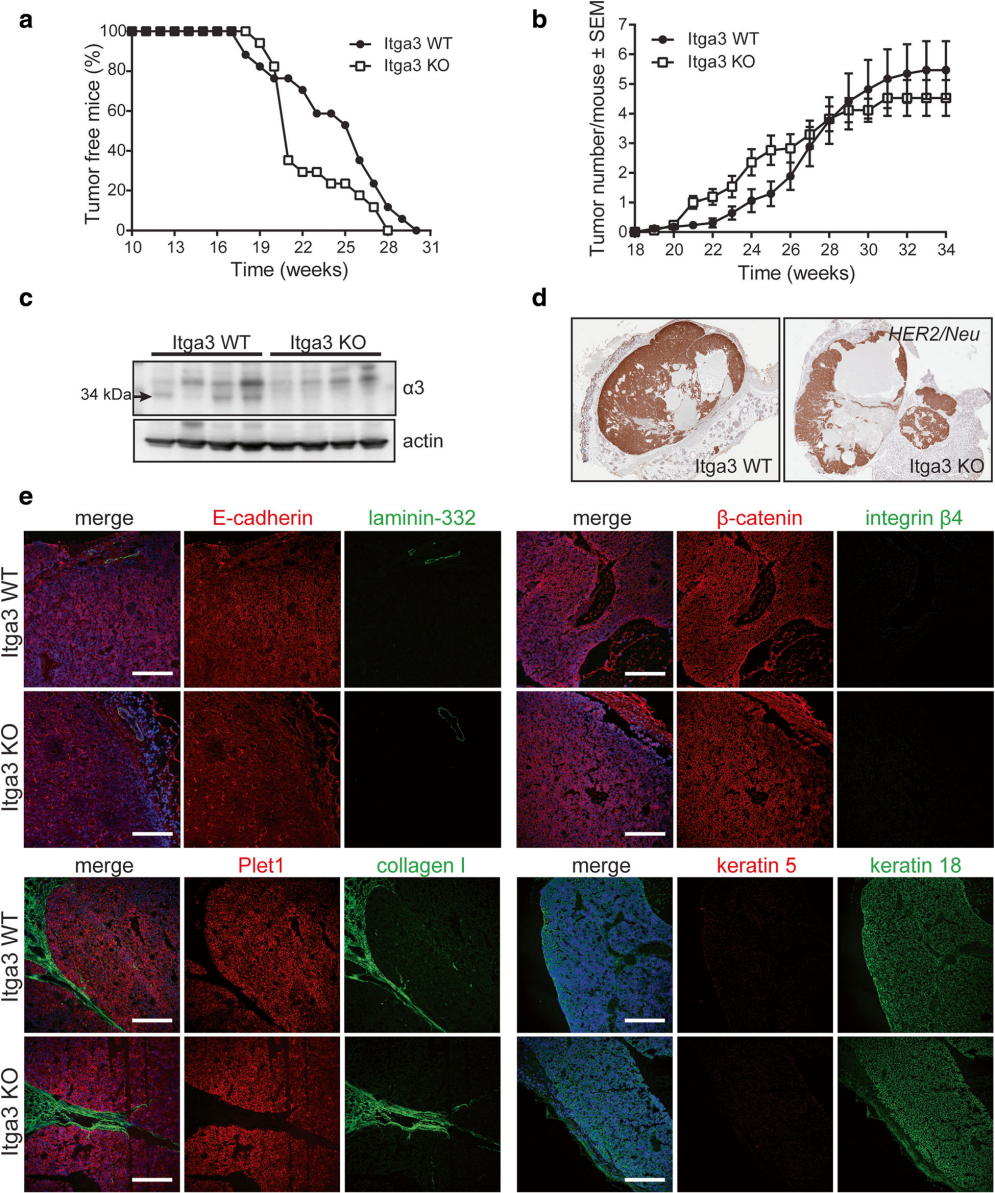
Integrin α3β1 is not needed for the onset of HER2-driven mammary tumorigenesis. **a** Kaplan-Meier plots of tumor-free survival in Itga3 KO and WT mice show that first palpable tumors could be detected at comparable age in both genotypes. **b** The number of tumors, measured macroscopically over time, was similar between Itga3 KO and WT mice (*n* = 17). **c** The western blot of four randomly selected, representative tumors isolated from Itga3 KO and WT mice shows a clear deletion of α3 in Itga3 KO mice. **d** Representative images of immunohistochemical staining for HER2/Neu in Itga3 KO and WT tumors show its strong expression, which was observed in all the analyzed tumors. **e** Representative images of immunofluorescent staining show the absence of basal markers β4, keratin 5, and laminin-332, which is also the main ligand for integrin α3β1. E-cadherin, β-catenin, Plet1, collagen I, and keratin 18 were strongly expressed in all tumors. No difference was observed between Itga3 KO and WT mice. Scale bar, 200 μm

HER2-mediated tumor onset and tumor number were not affected by the absence of integrin α3. First palpable tumors could be detected at day 126 in Itga3 WT and at day 133 in Itga3 KO group (Fig. 1a). On average, Itga3 WT and KO mice had developed 5.3 and 4.6 palpable tumors, respectively, when they were sacrificed at the defined humane endpoint (Fig. 1b). Histological analysis of the tumors revealed that mice developed two types of mammary adenocarcinomas, i.e., solid and cystic/hemorrhagic tumors (Additional file 1: Figure S1a), which were equally represented in both groups (Additional file 1: Figure S1b). The absence of α3 protein expression in the tumors of Itga3 KO group was confirmed by western blot analysis (Fig. 1c). Furthermore, immunohistochemistry showed high levels of expression of HER2/*Neu* (Fig. 1d), confirming their origin from cells driven by the expression of this oncogene. Tumors also showed high levels of E-cadherin, β-catenin, Plet1, and keratin 18, which in combination with the absence of basal markers integrin β4, Laminin-332, and keratin 5 indicates their luminal origin [35–37]. Abundant collagen I was observed in tumor stroma. No differences in the distribution and/or expression of these markers could be observed between tumors isolated from Itga3 WT or Itga3 KO mice (Fig. 1e). Together, these data show that α3 does not play an obvious role in the initial development and characteristics of HER2-dependent tumors in vivo*.*

### The absence of integrin α3 promotes HER2-dependent tumor growth and metastases formation

**Fig. 2:**
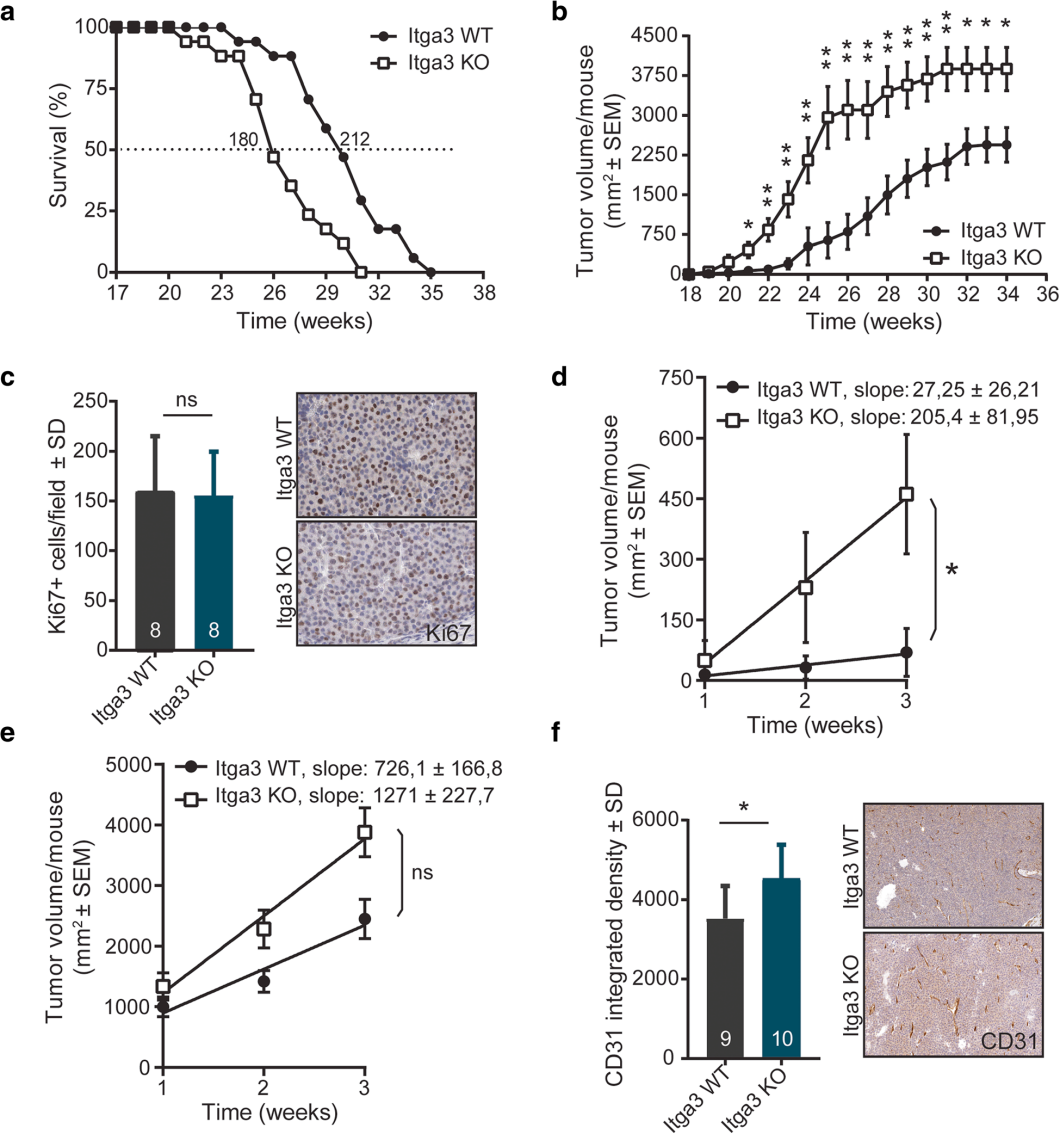
The absence of integrin α3 promotes HER2-dependent tumor growth and vascularization. **a** Survival Kaplan-Meier plots comparing Itga3 KO and WT mice. The median age when half of the mice per group needed to be sacrificed was 180 days for Itga3 KO mice and a month later (212 days) for Itga3 WT mice (*n* = 17). **b** Itga3 KO mice developed significantly bigger tumors from the age of 21 weeks on (*n* = 17; unpaired *t* test, *P < 0.05, **P < 0.005). **c** No difference in the number of proliferating Ki67-positive tumor cells was observed between Itga3 KO and WT mice. Left—Ki67-positive cells were counted in five fields of randomly selected tumors from eight mice per genotype (unpaired *t* test, *P* = 0.7168). Right—representative tumor images of immunohistochemical staining for Ki67. **d**, **e** Plots showing the fitted linear regression of average tumor sizes during **d** first and **e** last 3 weeks of tumor growth per mouse. The slopes of trend lines are significantly different during the first weeks of tumorigenesis, showing an increased growth rate in Itga3 KO mice. Such difference is not observed during the last 3 weeks of tumor growth before mice were sacrificed (*n* = 17, unpaired *t* test, *P < 0.05). **f** Vascularity of tumors, quantified as integrated density of CD31 stained samples (left), was increased in Itga3 KO mice. Five fields of randomly selected tumors from nine WT and ten KO mice were analyzed (unpaired *t* test, *P* = 0.0176). Right—representative tumor images of immunohistochemical staining for CD31

Despite the similar tumor onset in both groups, the survival of Itga3 KO mice was reduced compared to the WT group (Fig. 2a). In line with this, tumor volume was significantly increased in the absence of α3 (Fig. 2b). Interestingly, analysis of Ki67-positive cells in solid areas of Itga3 KO and WT tumors, isolated at the time the mice had to be sacrificed, showed no significant differences in the number of proliferating cells (Fig. 2c). Similarly, there were no differences in the number of apoptotic cells between Itga3 WT and Itga3 KO mice, as detected by cleaved Caspase-3 staining in solid and cystic adenocarcinomas (Additional file 1: Figure S1c). To investigate whether the differences in tumor volume between Itga3 KO and WT mice originated from different growth rates during the first weeks of tumor formation (i.e., in the early stages of oncogenesis), we determined the slopes of trend lines of average tumor size per mouse for both Itga3 KO and WT groups during the first 3 weeks after tumors were detected (Fig. 2d) and the last 3 weeks before individual mouse had to be sacrificed (Fig. 2e). Comparison of the slopes of the growth trend lines showed that Itga3 KO compared to WT tumors exhibited faster volumetric growth at the onset of tumorigenesis, whereas their growth rate during the last 3 weeks before the final time point was comparable. This could explain the differences in tumor volume, yet similar cell proliferation and apoptosis of the analyzed Itga3 KO and WT tumors at the time of sacrifice. Furthermore, histological analysis of blood vessel density in solid tumors, determined by measuring CD31-positive areas, showed an increased vascularization of Itga3 KO compared to WT tumors (Fig. 2f), which could offer a further explanation for the larger tumors in the Itga3 KO mice.

**Fig. 3:**
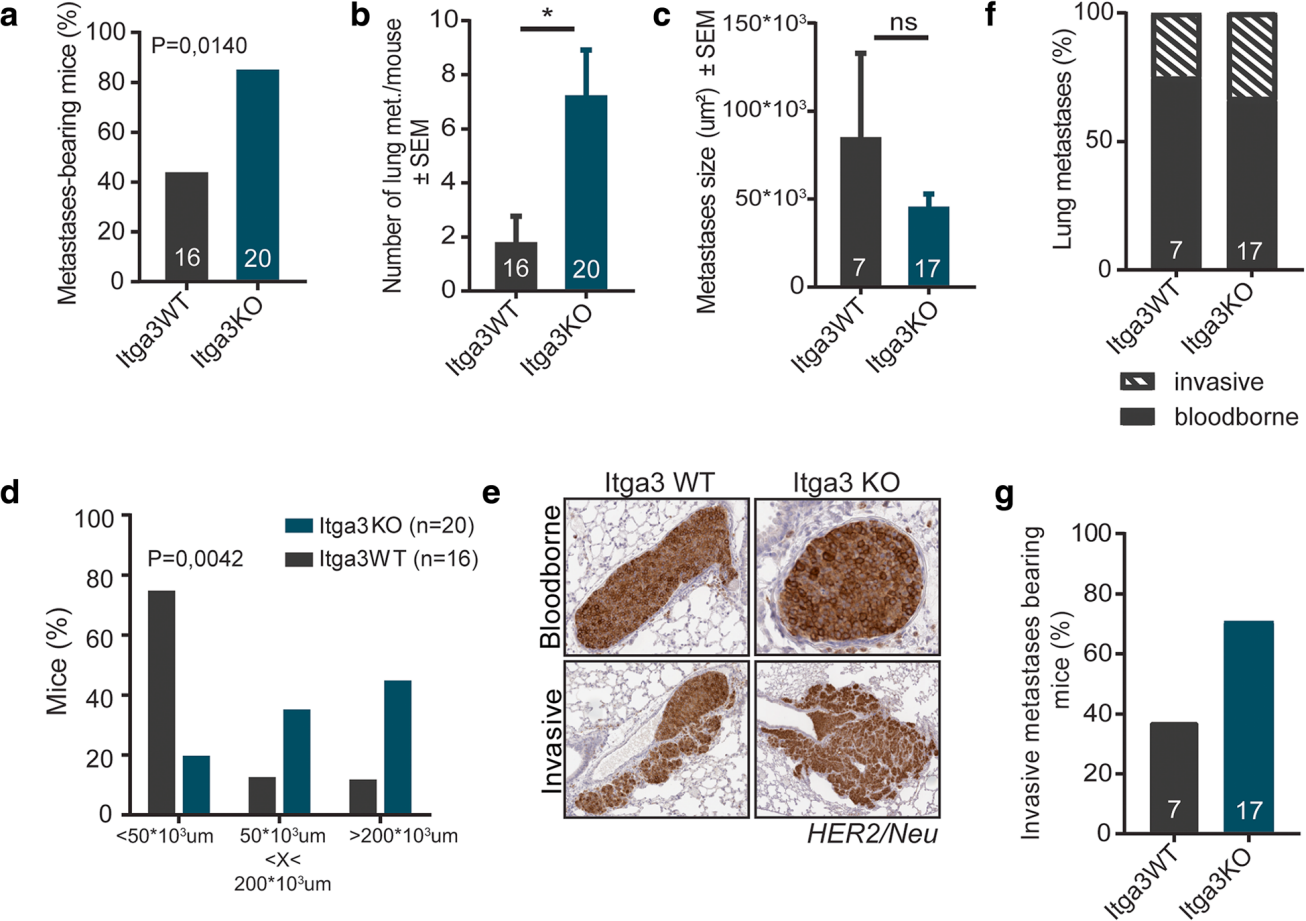
The absence of integrin α3 increases the invasiveness of HER2-driven tumors. **a** Significantly more Itga3 KO compared to WT mice developed lung metastasis (chi-square (Fisher’s exact) test). **b** The number of metastasis, counted in H&E-stained sections of the lungs, was significantly increased in Itga3 KO mice (unpaired *t* test, *P* = 0.0119). **c** Metastases were of similar sizes in Itga3 KO and WT mice (unpaired *t* test, *P* = 0.2222). **d** Itga3 KO compared to WT mice had a significantly larger total metastatic burden, i.e., total metastatic area in the lungs (chi-square test). **e** Representative images of invasive and blood-borne metastasis in the lungs of Itga3 KO and WT mice, stained for HER2/Neu. **f** The percentage of blood-borne and invasive out of total pulmonary metastases shows a small increase in the number of invasive metastases in Itga3 KO mice. **g** The percentage of invasive metastasis-bearing mice per genotype out of the total number of metastases-bearing mice. Almost twice as many Itga3 KO as WT mice displayed invasive metastases

The analysis of the organs of mice at the final time point showed the presence of metastases only in the lungs, which is common in MMTV-cNeu mice [38]. Pulmonary metastasis occurred in both Itga3 KO and WT mice, but while metastases were detected only in 40% of the Itga3 WT mice, nearly all the Itga3 KO mice had pulmonary lesions (Fig. 3a). Furthermore, the Itga3 KO mice had a significantly higher number of metastases (Fig. 3b), which, despite the comparable average size of metastases between both groups (Fig. 3c), resulted in significantly increased metastatic burden in Itga3 KO, compared to WT mice (Fig. 3d). HER2/*Neu*-positive lung metastases could be observed within the blood vessels (blood-borne metastases) or escaping the vasculature and invading the surrounding lung parenchyma (invasive metastatic lesions). Both types of metastases could be detected in Itga3 WT and KO mice (Fig. 3e). A moderate increase in the percentage of invasive lesions out of their total number was observed in the Itga3 KO mice (Fig. 3f). Importantly, invasive metastatic lesions were detected in 37.5% of metastasis-bearing Itga3 WT mice, whereas in the Itga3 KO mice, this number raised to 70% (Fig. 3g).

In order to investigate whether the absence of α3 promotes tumor progression and invasiveness through changes in the downstream signaling of HER2, we have analyzed the primary tumors and metastases for the activation of protein kinase B (pAkt), the mitogen-activated protein kinase 1 and 2 (pErk1/2), and eukaryotic translation initiation factor 4E-binding protein 1 (p4E-BP1), a downstream target of mTOR signaling pathway [5]. No differences were observed between Itga3 WT and KO mice (Additional file 2: Figure S2), suggesting that major alterations in MAPK and phosphoinositide 3-kinase (PI3K) signaling do not lie at the basis of the metastasis-promoting effect of the absence of α3β1. Together, these findings demonstrate that the absence of α3 promotes tumor growth and vascularization and strongly increases the invasive and metastatic potential of HER2-driven breast cancer, resulting in reduced overall survival of Itga3 KO mice.

### Reduced expression of α3 is associated with increased invasiveness of HER2+, but not of triple-negative human mammary carcinoma cells

**Fig. 4:**
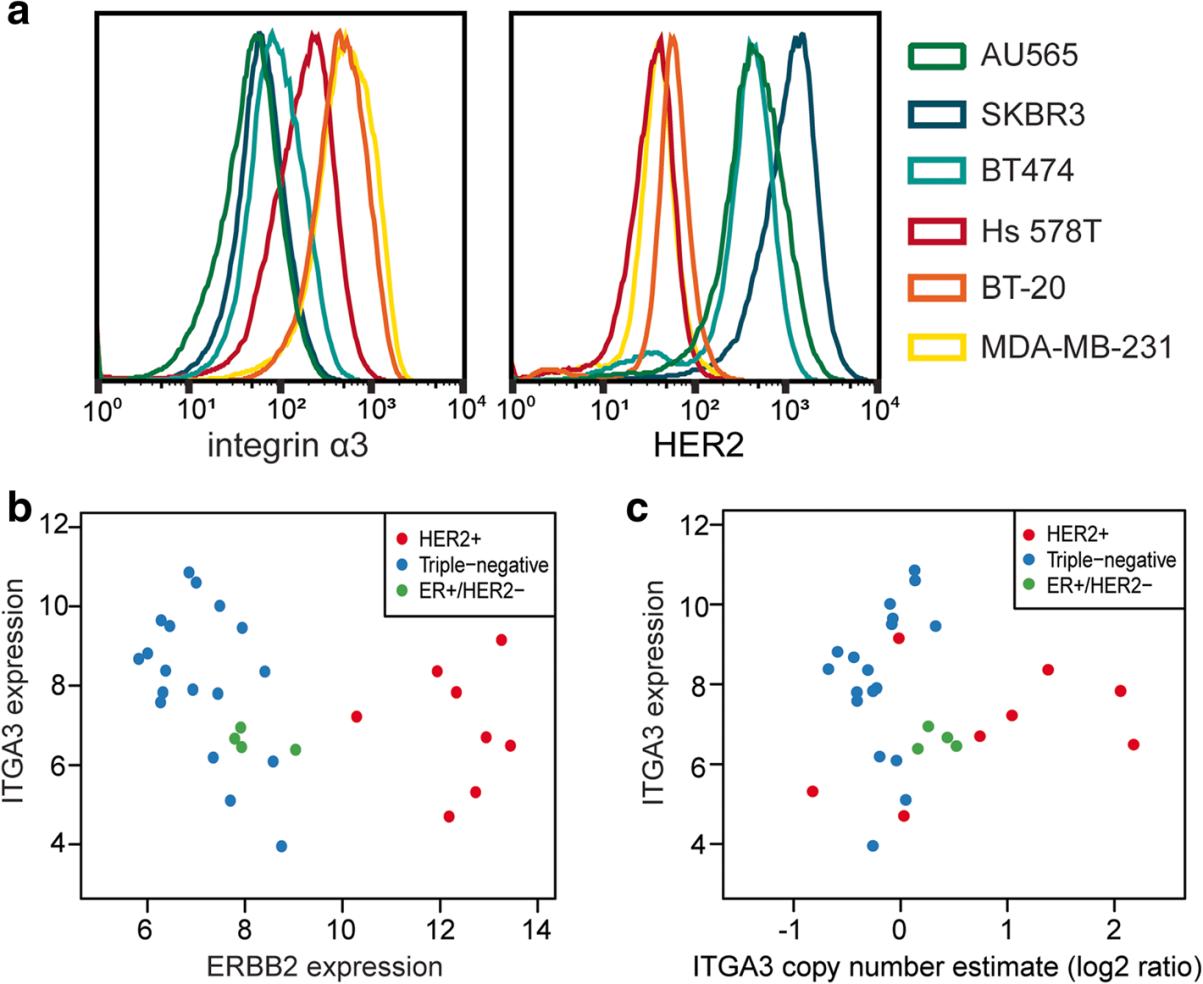
Decreased ITGA3 expression in HER2+, compared to triple-negative human carcinoma cells. **a** Flow cytometry histograms of signal intensity for α3 (left) and HER2 (right) staining of six human mammary carcinoma cell lines show lower surface expression of α3 in HER2+ cells. 50 × 10^3^ cells were analyzed per experiment; representative graphs of three independent experiments. **b** Scatter plot of ITGA3 and ERBB2 expression for HER2+ and triple-negative-enriched breast cancer panel [22] shows a negative correlation between ITGA3 and ERBB2 expression (Spearman’s rho − 0.46, *P* = 0.01, *n* = 30). **c** Scatter plot of ITGA3 gene copy number estimates against ITGA3 expression for HER2+ and triple-negative-enriched breast cancer panel [22]. Despite ITGA3 amplification in several HER2+ cell lines, their expression of ITGA3 remains relatively low

Next, we investigated whether reduced expression of α3 increases the invasiveness of human mammary carcinoma cells. The surface expression of α3 was analyzed in three established human HER2+ (SKBR3, AU565, and BT474) and three triple-negative (MDA-MB-231, BT-20, and Hs 578T) mammary carcinoma cell lines using flow cytometry. The surface levels of α3 were reproducibly lower in all three HER2+ cell lines (Fig. 4a), which was confirmed by western blot analysis of total cell lysates (Additional file 3: Figure S3a). This is in line with previous observations of the α3 protein levels in MDA-MB-231 and SKBR3 cells [20] and suggests that downregulation of α3β1 integrin is associated with HER2-driven mammary tumorigenesis and tumor progression. Available RNA sequencing data from a panel of HER2+ and triple-negative-enriched breast cancer cell lines [22] also showed a negative correlation between ERBB2 and ITGA3 expression (Fig. 4b), although no such clear correlation could be observed in larger and more diverse breast cancer cell line panel [32, 33], indicating that the regulation of α3β1 expression could be breast cancer cell type-dependent (Additional file 3: Figure S3b). In line with this, luminal-like breast cancer cell lines form a clear cluster of low ITGA3 expression in both datasets (Additional file 3: Figure S3c and S3d). Interestingly, both datasets showed relatively low ITGA3 expression in several HER2+ cell lines despite ITGA3 gene amplification (Fig. 4c, Additional file 3: Figure S3e). Together, these data indicate that HER2-driven, luminal-like breast cancer cells exhibit lower ITGA3 expression than triple-negative breast cancer cells.

**Fig. 5:**
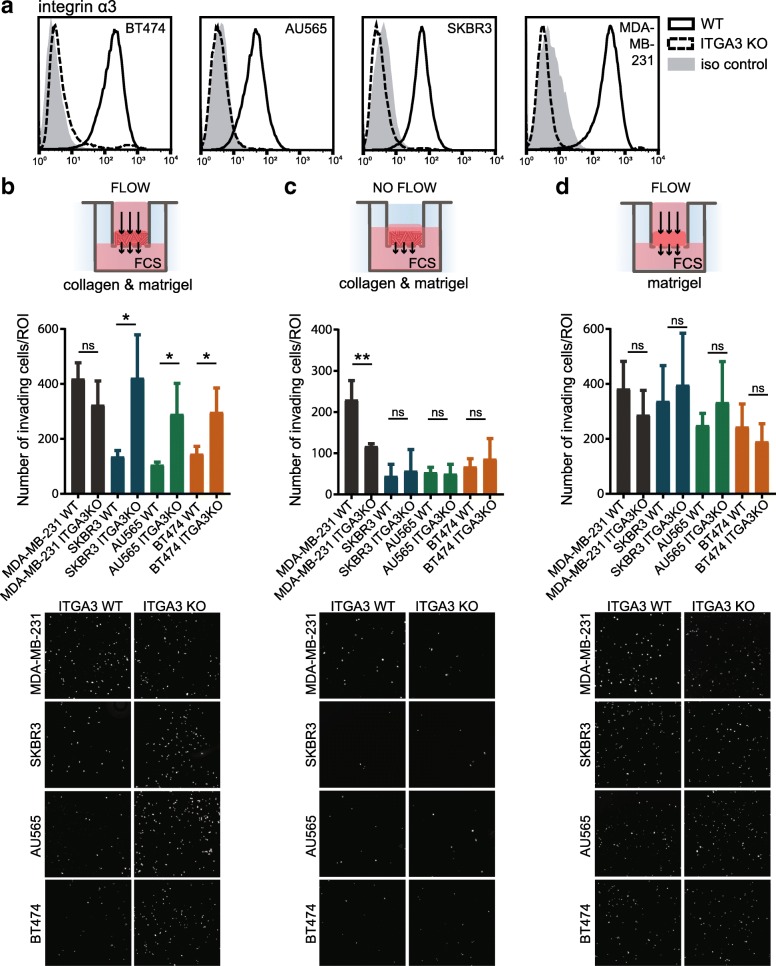
α3 reduction causes increased invasiveness of HER2+, but not triple-negative human carcinoma cells. **a** Flow cytometry histograms of signal intensity of α3 show successful α3 deletion in AU565, SKBR3, BT474, and MDA-MB-231cells. **b**–**d** Invasion assays through gel-coated (Matrigel-collagen I or Matrigel only) membrane. Bottom—representative images of the part of the membrane, showing DAPI-stained nuclei of invading cells. Top—analysis of the experiments, performed in duplicate and repeated three times. (mean ± SD, unpaired *t* test, *P < 0.05, **P < 0.005) **b** The reduction of α3 increases the invasiveness of HER2+ carcinoma cells through the mixture of collagen I and Matrigel under interstitial fluid flow conditions. **c** Under static conditions, HER2+ cells show strongly reduced and α3-independent invasion. Contrary, the invasion of triple-negative MDA-MB-231 is α3-dependent. **d** The absence of collagen I causes an increased invasion of α3WT HER2+ carcinoma cells, resulting in similar levels of invasion between α3KO and WT cells

To assess whether α3β1 influences the invasive potential of HER-driven human mammary carcinoma cells, we generated SKBR3, AU565, and BT474 ITGA3 KO cell lines by CRISPR/Cas9 technology (Fig. 5a). No difference in HER2 levels or downstream Akt signaling was observed between ITGA3 WT and KO cells (Additional file 4: Figure S4a). For comparison, we have also deleted α3 in triple-negative MDA-MB-231 cells (Fig. 5a). Cell invasion was assessed in Transwell chambers with membranes coated with a mixture of Matrigel and collagen I, which was abundantly present around primary tumors in our in vivo model (Fig. 1e), and using 10% FCS as a chemoattractant. To mimic high interstitial fluid flow in tumors, caused by angiogenesis and increased vascular permeability, pressure was applied to the tumor cells by an overlying column of a serum-free medium in the upper chamber [39]. To control for the flow rates in the different experiments, we measured the volume of the medium that has passed through the gel and membrane (Additional file 4: Figure S4b). As shown in Fig. 5b, there were no significant differences in the number of invading cells for triple-negative MDA-MB-231 ITGA3 KO and WT cells. In contrast and consistent with in vivo observations, all HER2+ cell lines exhibited a significantly higher invasion in the absence of α3 (Fig. 5b). Together, these data suggest that the absence of α3 promotes migration of HER2+, but not triple-negative carcinoma cells in an environment with interstitial fluid pressure.

### The invasion-suppressing function of α3 in HER2+ cells depends on the extracellular matrix composition and the interstitial fluid flow

We further investigated whether the microenvironment, such as interstitial fluid flow and extracellular matrix composition, plays a role in the increased invasive potential of HER2+ carcinoma cells lacking α3. First, we allowed cells to invade through the membrane, coated with a mixture of Matrigel and collagen I in the absence of fluid flow. Under these conditions, the deletion of α3 in MDA-MB-231 cells significantly impaired their invasive potential, which is in line with previous reports [20, 21]. In contrast, all three HER2-driven carcinoma cell lines exhibited very low invasive potential, with no obvious differences between the ITGA3 KO and WT cells (Fig. 5c). Next, we assessed the role of collagen I in the extracellular matrix by performing the invasion assays under interstitial fluid flow, but with membranes, coated only with Matrigel. As before, the flow rates through the coated membranes were similar for the different cell lines (Additional file 4: Figure S4c). However, different from that observed with the membrane coated with a collagen-Matrigel mixture, no significant differences in cell invasion were observed between the ITGA3 KO and WT HER+ cells (Fig. 5d). Therefore, the increased invasive potential of ITGA3 KO HER2+ carcinoma cells strongly depends on the presence of the interstitial fluid flow and a collagen I-rich extracellular matrix.

**Fig. 6:**
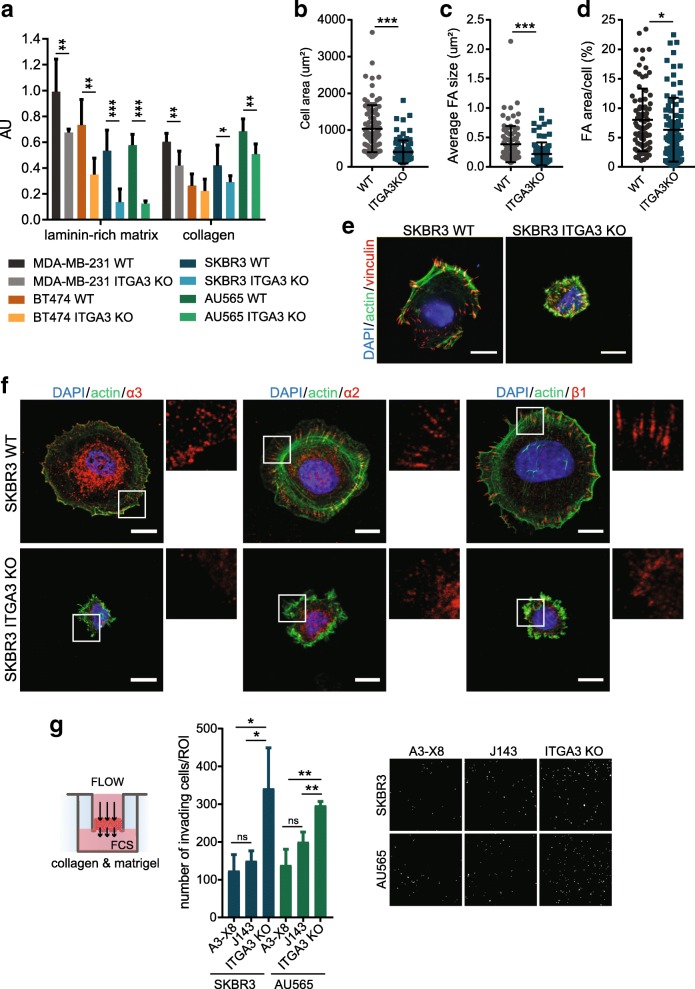
α3-mediated changes in collagen adhesion and FA formation. **a** Short-term adhesion assays of HER− MDA-MB-231 and HER2+ BT474, AU565, and SKBR3 on laminin-rich matrix and collagen I. Experiments were performed in triplicate and repeated three times (mean ± SD, unpaired *t* test, *P < 0.05, **P < 0.005, ***P < 0.0001). **b** α3KO SKBR3 show significantly decreased cell area, **c** reduced size of focal adhesions, and **d** reduced total adhesion area per cell after 30 min of adhesion to collagen I. Experiments were performed three times with 30 cells analyzed per experiment (total *n* = 90, unpaired *t* test, *P < 0.05, **P < 0.005, ***P < 0.0005). **e** Representative images of SKBR3 ITGA3 KO and WT cell, used for quantifications (**b**–**d**), stained for actin and vinculin (scale bar, 10 μm). **f** Representative images of SKBR3 ITGA3 KO and WT cells, allowed to adhere to collagen I-coated coverslips for 30 min and stained for actin and integrins α3, α2, or β1. ITGA3 KO cells show reduced clustering of integrins in adhesion complexes. Note that α3 and α2 signals were enhanced by biotin-conjugated secondary antibody, resulting in unspecific biotin staining in the center of the cell (Additional file 4: Figure S4b) (scale bar, 10 μm). **g** Invasion assays through the mixture of collagen I and Matrigel-coated membrane. The reduction of α3 increases invasiveness of HER2+ carcinoma cells under interstitial fluid flow conditions, which cannot be recapitulated with blocking adhesion of α3 to laminin by addition of function-blocking J143 antibody (A3-X8: control non-blocking antibody). Left—analysis of the experiments, performed in duplicate and repeated three times (mean ± SD, unpaired *t* test, *P < 0.05, **P < 0.005). Right—representative images of the part of the membrane, showing DAPI-stained nuclei of invading cells

Interestingly, the number of ITGA3 WT HER2+ cells invading the Matrigel was increased to a level comparable to that of the invading ITGA3 KO cells, which is similar in both collagen-Matrigel and Matrigel gels under fluid flow conditions (Fig. 5b, d). This finding made us wonder whether α3β1 contributes to the adhesion of cells to collagen, leading to faster “passive” migration of ITGA3 KO cells through the collagen I-rich extracellular matrix when fluid pressure is applied. To investigate the effect of α3 deletion on the adhesion of cells to collagen, we performed short-term adhesion assays. HER2+ SKBR3, AU565, and BT474, and triple-negative MDA-MB-231 cells were seeded on matrices rich in laminin-332 or collagen I and allowed to adhere for 30 min, after which the number of adherent cells was quantified. As expected, the adhesion of all ITGA3 KO cell lines to laminin-332-rich matrices was strongly reduced. Deletion of ITGA3 also resulted in a moderate but significant decrease in adhesion of triple-negative MDA-MB-231 and HER2+ SKBR3 and AU565 (but not BT474) cells to collagen I (Fig. 6a). Furthermore, the spread area and size of FAs of ITGA3 KO SKBR3 cells were significantly reduced compared to WT cells (Fig. 6b–e). No changes in the surface expression of the collagen receptors α1β1 and α2β1 integrin were observed in ITGA3 KO cells (Additional file 5: Figure S5a). However, IF staining of SKBR3 ITGA3 KO cells showed reduced clustering of integrin α2 and its binding partner β1 to FAs during the first 30 min of adhesion to collagen I (Fig. 6f). This suggests that α3β1 may promote adhesion and spreading of cells on collagen by promoting the clustering of collagen-binding integrins and thus the formation of FAs. In line with this, the invasion-suppressing effect of α3β1, observed during the invasion of HER2+ cells through a Matrigel/collagen I mixture under fluid flow conditions, was not dependent on the adhesive activity of this integrin. No clear differences in the invasion were observed between SKBR3 and AU565 cells, treated with α3 function-blocking antibody J143, which prevents the ligation of α3β1 by laminin, and non-blocking control antibody A3-X8 [26]. As observed before, the depletion of α3 significantly increased the invasion of both cell lines (Fig. 6g, Additional file 5: Figure S5c).

Together, our data suggest that the reduction of α3 allows HER2-driven carcinoma cells to migrate and invade faster through highly vascularized, collagen I-rich tumor stroma, which could be partially explained through the decreased adhesion to collagen I.

## Discussion

In this study, we show that loss of the α3β1 integrin promotes HER2-driven luminal-type of breast cancer in vivo. In this tumor model, the initial steps of the tumorigenesis developed independently of the presence of α3β1; however, α3β1-depleted tumors grew bigger, were highly vascularized, and, importantly, displayed strongly increased metastatic potential.

The studies, describing the essential role of α3β1 in tumor initiation, usually investigated epithelial tumors of basal nature, i.e., originating from epithelial cells anchored to a laminin-rich basement membrane, such as non-melanoma skin tumors and ovarian cancers [40, 41]. Similarly, α3β1 supports tumor initiation and progression in basal-type breast cancer [18, 19, 42]. Such pivotal role of α3β1 in basal-type tumors is often linked to its ability to support oncogenic MAPK signaling upon its ligation by laminin-332 [43], which may be crucial for the proliferation and survival of tumor cells in early stages when cells still depend on adhesion for their proliferation. This mechanism has indeed been observed in an in vivo mouse model of the basal-type breast cancer, in which α3β1 promotes proliferation and survival of tumor cells through activation of the FAK-PAK1-ERK1/2 signaling pathway [18]. Furthermore, the ability of α3β1 to support and sustain the activation of signaling pathways upon its ligation to laminin might be necessary also during the later stages of breast cancer when cells acquire additional mutations, i.e., during the epithelial-mesenchymal transition and invasion [19, 42].

In contrast with this evident pro-tumorigenic function of α3β1 when bound to laminin, its laminin-independent role, such as described in our model, remains more elusive. We observed no clear differences in the activation of pro-survival and proliferation-promoting pathways between Itga3 KO and WT mice. The downstream signaling of HER2 that drives the formation and progression of tumors therefore appears to be independent of α3β1, enabling normal tumor onset and initial tumor development in Itga3 KO mice. This finding is seemingly in contradiction with a previously reported study by Novitskaya et al. [44], showing that α3β1 (in complex with CD151) supports the growth of SKBR3 and BT474 cells in Matrigel by promoting the homodimerization and activation of HER2 via inhibition of RhoA. However, the downregulation of α3 only affected the phosphorylation and homodimerization of HER2, whereas Akt signaling (as we confirm in this study) and phosphorylation of HER3, another member of epidermal growth factor receptor family, were unperturbed. Therefore, it seems likely that in their model, the majority of pro-survival and proliferative signaling came from the dimerization of HER2 with HER3, i.e., the most potent mitogenic signaling dimer in the family [45]. Furthermore, it has been shown that HER2 activates pro-metastatic RhoA and RhoC in vivo and in vitro [46].

Despite the similar proliferation rate and the fact that we observed no differences in HER2-driven signaling events between Itga3 KO and WT mice at the time when mice were sacrificed, the absence of α3β1 promoted tumor growth during the early stage of tumorigenesis. One of the obstacles that tumor cells must overcome during this stage of tumor mass accumulation is the absence of vascularity, and consequent hypoxic environment and lack of nutrients. It is possible that the increased angiogenesis that we observed in Itga3 KO mice in the late stage of tumorigenesis could have contributed to the differences in tumor growth rate during the first weeks of fast tumor mass accumulation when earlier and/or increased vessel formation would likely result in strong growth advantage. In line with this, it has been observed that the reduction of α3β1 in prostate carcinoma cells promoted their proliferation via changes in the interaction between tumor and stromal cells [47].

High vascularization and vascular permeability of tumors lead to interstitial fluid flow that is increased compared to normal tissues, promoting the dissemination of cells and metastases formation [48]. Therefore, an increased angiogenesis of Itga3 KO tumors might already (partially) explain their faster progression and increased invasion. Furthermore, our data show that fluid flow and collagen I-rich extracellular matrices play a crucial role in an increased invasive potential of ITGA3 KO HER2+ SKBR3, AU565, and BT474 cells. Such increased invasiveness can be partially explained by their reduced adhesion to collagen I, an extracellular matrix component that is abundant in the mammary tumor stroma [49–51]. In line with this, it has been shown that deletion of the collagen receptor integrin α2β1 increases intravasation, but not extravasation of tumor cells, which results in strongly increased metastases formation in HER2/Neu-overexpressing mouse model [52]. The reduced adhesion to collagen I that we have observed in MDA-MB-231, SKBR3, and AU565 ITGA3 KO mammary carcinoma cells is not due to the changes in the α2β1 expression, but likely due to the reduced clustering of α2β1. Indeed, the absence of α3 affected the size and area of FAs during initial adhesion of SKBR3 cells to the collagen I. In line with this, it has been well documented that the presence of α3β1 can affect the formation and/or dynamics of other adhesion complexes [53, 54]. Furthermore, clustering of integrins is connected to their increased activity and therefore increased outside-in signaling, which, among others, leads to changes in the actomyosin contractility [55].

However, no α3-dependent differences in adhesion to collagen I were observed in HER2+ BT474 cells, even though they showed an increased invasiveness in collagen I-rich matrix under flow conditions upon α3 depletion. Therefore, α3-mediated changes in cell adhesion cannot fully explain the observed differences in the invasion potential. One important difference between Matrigel and the mixture of collagen and Matrigel is their degree of stiffness. The addition of collagen I to Matrigel results in an increased stiffness, which can strongly impact the invasion and migration through alterations in mechanosensing and mechanotransduction [56, 57]. Furthermore, collagen I-rich, stiff and dense tissue is a known risk factor for developing breast carcinoma and metastases [51, 58]. In such environment, Rho-driven actomyosin contractility plays an important role in migration and invasion [51, 57, 59]. As already mentioned, the downregulation of Rho by α3β1-CD151 complexes is well established [8, 12]; therefore, it is possible that α3 depletion promotes invasion also through increased Rho activity of cells in vivo and in vitro.

Finally, our experiments confirmed the previously reported pro-invasive role of α3 in MDA-MB-231 triple-negative breast cancer [19–21]. Invasion assays performed in the presence or absence of interstitial fluid flow demonstrated different invasive behaviors of triple-negative MDA-MB-231, compared to HER2+ AU565, SKBR3, and BT474 cells even when α3 was not deleted. Furthermore, expression analysis of two different datasets of breast cancer cell lines suggests cancer subtype-dependent regulation of α3 expression, with its downregulation in invasive HER2+ luminal-like carcinoma cells. As it is increasingly evident that the existing classifications of breast cancer subtypes often overlap and struggle to classify the heterogeneity of the disease [60], it may be naïve to expect that the function of α3β1 in breast cancer can only be predicted by the HER2 status. However, striving to understand the impact of α3β1 and other similar markers under specific and defined conditions of the disease, such as HER2-overexpression, the composition of the extracellular matrix and luminal cell origin can help us towards its better clinical definition and consequently more efficient treatment strategies.

## Conclusion

This study shows that the downregulation of α3β1 in HER2-driven mouse model and in HER2+ human mammary carcinoma cells promotes tumor progression and invasiveness of the cells. It demonstrates that the collagen I-rich extracellular matrix and interstitial fluid flow define the invasive potential of α3-depleted HER2+ cells in vitro, implying that such environmental factors may mediate the invasiveness of cells in highly vascularized tumors in the absence of α3β1 and laminin-332. Importantly, the observed role of α3β1 in HER2-driven mammary tumorigenesis was not observed in triple-negative MDA-MB-231 cells, where the downregulation of α3β1 resulted in the opposite effect on invasiveness, which clearly demonstrates the tumor type-specific function of α3β1 in breast cancer.

## Additional files

Additional file 1: **Figure S1.** (a) Representative macroscopic and H&E-stained pictures of the two types of tumors detected: solid tumors with high cellularity and cystic tumors which consist of multiple large and small cysts/open areas filled with blood. Scale bar, 100 μm. (b) Quantification of solid vs cystic tumors Itga3 KO and WT mice determined by pathological analysis of excised tumors. No significant differences between both groups could be detected. (c) Immunohistochemical staining for cleaved-Cas3 marker of apoptosis in representative solid (top) and cystic (bottom) tumors of Itga3 KO and WT mice. No positive staining was observed in any of the analyzed solid tumors, and a comparable amount of apoptosis was detected in cystic tumors of Itga3 KO and WT mice. Scale bar, 100 μm. (TIF 4018 kb)

Additional file 2: **Figure S2.** Representative images of immunohistochemical staining of (a) primary tumors and (b) metastases of Itga3 KO and WT mice. No differences were observed in the activation of main HER2-mediated pathways, as seen by pAkt, pErk, and p4E-BP1 staining. Scale bars, 200 μm. (TIF 8790 kb)

Additional file 3: **Figure S3.** (a) Representative western blot (left) and quantification (right) of three separate experiments of whole cell lysates of triple-negative MDA-MB-231, BT-20, Hs 578T and HER2+ BT474, AU565, and SKBR3 mammary carcinoma cells. HER2-overexpressing cell lines exhibit strongly reduced levels of α3 protein. (b) Scatter plot showing a lack of correlation between ITGA3 and ERBB2 expression in CCLE breast cancer panel (Spearman’s rho − 0.17, *P* = 0.22, *n* = 51). (c-d) Scatter plots of ITGA3 and ERBB2 expression of breast cancer cell lines, classified as luminal-, basal-, and post-EMT-like show clustering of luminal-like cell lines to low ITGA3 expression: (c) HER2+ and triple-negative-enriched breast cancer panel [22] (*n* = 30). (d) CCLE breast cancer cell panel (*n* = 51). (e) Scatter plot of ITGA3 gene copy number estimates against ITGA3 expression for CCLE dataset. Despite ITGA3 amplification in several HER2+ cell lines, their expression of ITGA3 remains relatively low. (TIF 1468 kb)

Additional file 4: **Figure S4.** (a) Representative western blot (left) and quantification (right) of three separate experiments of whole cell lysates of WT and ITGA3 KO AU565 and SKBR3 mammary carcinoma cells. No differences in the levels of HER2 or in Akt signaling were observed between ITGA3 KO and WT cells. (b-c) Volume of medium, passing the (b) mixture of collagen I and Matrigel and (c) Matrigel only during the invasion assays under interstitial flow conditions (mean ± SD). No significant differences were observed (one-way ANOVA). (TIF 1500 kb)

Additional file 5: **Figure S5.** (a) Flow cytometry histograms of surface expression of collagen-binding integrins α1 and α2 and laminin-binding integrin α6 in ITGA3 KO and WT mammary carcinoma cells. (b) Representative image of SKBR3 ITGA3 WT cells, stained with biotin-conjugated secondary antibody as a background control (scale bar, 10 μm). (c) Volume of medium, passing the mixture of collagen I and Matrigel during the invasion assays under interstitial flow conditions and with the addition of α3-function blocking (J143) and control (A3-X8) antibodies (mean ± SD). No significant differences were observed (one-way ANOVA). (TIF 2113 kb)

## Abbreviations

4E-BP1: Eukaryotic translation initiation factor 4E-binding protein 1
AB: Antibody
Akt: Protein kinase B
ANOVA: Analysis of variance
CCLE: Cancer Cell Line Encyclopedia
DAPI: 4′,6-Diamidino-2-phenylindole
ER: Estrogen receptor
FA: Focal adhesion
FACS: Fluorescent activated cell sorting
HER2: Human epidermal growth factor receptor 2
IF: Immunofluorescence
IHC: Immunohistochemical analysis
MAPK, Erk1/2: Mitogen-activated protein kinase 1 and 2
MMTV: Mouse mammary tumor virus
PI3K: Phosphoinositide 3-kinase
WB: Western blot

## References

[1] Hynes NE, MacDonald G (2009). ErbB receptors and signaling pathways in cancer. Curr Opin Cell Biol.

[2] Muller WJ, Sinn E, Pattengale PK, Wallace R, Leder P (1988). Single-step induction of mammary adenocarcinoma in transgenic mice bearing the activated c-neu oncogene. Cell..

[3] Ursini-Siegel J, Schade B, Cardiff RD, Muller WJ (2007). Insights from transgenic mouse models of ERBB2-induced breast cancer. Nat Rev Cancer.

[4] Hynes NE, Lane HA (2005). ERBB receptors and cancer: the complexity of targeted inhibitors. Nat Rev Cancer.

[5] Fry EA, Taneja P, Inoue K (2017). Oncogenic and tumor-suppressive mouse models for breast cancer engaging HER2/neu. Int J Cancer.

[6] Eccles SA (2001). The role of c-erbB-2/HER2/neu in breast cancer progression and metastasis. J Mammary Gland Biol Neoplasia.

[7] Kennecke H, Yerushalmi R, Woods R, Cheang MCU, Voduc D, Speers CH (2010). Metastatic behavior of breast cancer subtypes. J Clin Oncol Off J Am Soc Clin Oncol.

[8] Stipp CS. Laminin-binding integrins and their tetraspanin partners as potential antimetastatic targets. Expert Rev Mol Med. 2010;12:e3. 10.1017/S1462399409001355. 10.1017/S1462399409001355 PMC2811424 20078909

[9] Lahlou H, Muller WJ (2011). β1-integrins signaling and mammary tumor progression in transgenic mouse models: implications for human breast cancer. Breast Cancer Res.

[10] Longmate W, DiPersio CM (2017). Beyond adhesion: emerging roles for integrins in control of the tumor microenvironment. F1000Research..

[11] Chen CS, Alonso JL, Ostuni E, Whitesides GM, Ingber DE (2003). Cell shape provides global control of focal adhesion assembly. Biochem Biophys Res Commun.

[12] Ramovs V, Te Molder L, Sonnenberg A. The opposing roles of laminin-binding integrins in cancer. Matrix Biol J Int Soc Matrix Biol. 2017;57–58:213–43. 10.1016/j.matbio.2016.08.007 27562932

[13] Berry MG, Gui GPH, Wells CA, Carpenter R (2004). Integrin expression and survival in human breast cancer. Eur J Surg Oncol.

[14] Aggarwal A, Al-Rohil RN, Batra A, Feustel PJ, Jones DM, DiPersio CM (2014). Expression of integrin α3β1 and cyclooxygenase-2 (COX2) are positively correlated in human breast cancer. BMC Cancer.

[15] Gui GP, Wells CA, Browne PD, Yeomans P, Jordan S, Puddefoot JR (1995). Integrin expression in primary breast cancer and its relation to axillary nodal status. Surgery..

[16] Pignatelli M, Hanby AM, Stamp GW (1991). Low expression of beta 1, alpha 2 and alpha 3 subunits of VLA integrins in malignant mammary tumours. J Pathol.

[17] Romanska HM, Potemski P, Krakowska M, Mieszkowska M, Chaudhri S, Kordek R, et al. Lack of CD151/integrin α3β1 complex is predictive of poor outcome in node-negative lobular breast carcinoma: opposing roles of CD151 in invasive lobular and ductal breast cancers. Br J Cancer. 2015;113(9):1350–7. 10.1038/bjc.2015.344 PMC4815791 26418423

[18] Cagnet S, Faraldo MM, Kreft M, Sonnenberg A, Raymond K, Glukhova MA. Signaling events mediated by α3β1 integrin are essential for mammary tumorigenesis. Oncogene. 2014;33(34):4286–95. 10.1038/onc.2013.391 24077284

[19] Zhou B, Gibson-Corley KN, Herndon ME, Sun Y, Gustafson-Wagner E, Teoh-Fitzgerald M (2014). Integrin α3β1 can function to promote spontaneous metastasis and lung colonization of invasive breast carcinoma. Mol Cancer Res.

[20] Morini M, Mottolese M, Ferrari N, Ghiorzo F, Buglioni S, Mortarini R (2000). The alpha 3 beta 1 integrin is associated with mammary carcinoma cell metastasis, invasion, and gelatinase B (MMP-9) activity. Int J Cancer.

[21] Mitchell K, Svenson KB, Longmate WM, Gkirtzimanaki K, Sadej R, Wang X (2010). Suppression of integrin alpha3beta1 in breast cancer cells reduces cyclooxygenase-2 gene expression and inhibits tumorigenesis, invasion, and cross-talk to endothelial cells. Cancer Res.

[22] Jastrzebski K, Thijssen B, Kluin RJC, de Lint K, Majewski IJ, Beijersbergen RL (2018). Integrative modeling identifies key determinants of inhibitor sensitivity in breast cancer cell lines. Cancer Res.

[23] Cong L, Ran FA, Cox D, Lin S, Barretto R, Habib N (2013). Multiplex genome engineering using CRISPR/Cas systems. Science..

[24] Giltay JC, Brinkman HJ, Modderman PW, von dem Borne AE, van Mourik JA (1989). Human vascular endothelial cells express a membrane protein complex immunochemically indistinguishable from the platelet VLA-2 (glycoprotein Ia-IIa) complex. Blood..

[25] Fradet Y, Cordon-Cardo C, Thomson T, Daly ME, Whitmore WF, Lloyd KO (1984). Cell surface antigens of human bladder cancer defined by mouse monoclonal antibodies. Proc Natl Acad Sci U S A.

[26] Weitzman JB, Pasqualini R, Takada Y, Hemler ME (1993). The function and distinctive regulation of the integrin VLA-3 in cell adhesion, spreading, and homotypic cell aggregation. J Biol Chem.

[27] Sonnenberg A, Janssen H, Hogervorst F, Calafat J, Hilgers J (1987). A complex of platelet glycoproteins Ic and IIa identified by a rat monoclonal antibody. J Biol Chem.

[28] Raymond K, Richter A, Kreft M, Frijns E, Janssen H, Slijper M (2010). Expression of the orphan protein Plet-1 during trichilemmal differentiation of anagen hair follicles. J Invest Dermatol.

[29] Rueden CT, Schindelin J, Hiner MC, DeZonia BE, Walter AE, Arena ET (2017). ImageJ2: ImageJ for the next generation of scientific image data. BMC Bioinformatics.

[30] Schindelin J, Arganda-Carreras I, Frise E, Kaynig V, Longair M, Pietzsch T (2012). Fiji: an open-source platform for biological-image analysis. Nat Methods.

[31] Delwel GO, de Melker AA, Hogervorst F, Jaspars LH, Fles DL, Kuikman I (1994). Distinct and overlapping ligand specificities of the alpha 3A beta 1 and alpha 6A beta 1 integrins: recognition of laminin isoforms. Mol Biol Cell.

[32] Barretina J, Caponigro G, Stransky N, Venkatesan K, Margolin AA, Kim S (2012). The Cancer Cell Line Encyclopedia enables predictive modelling of anticancer drug sensitivity. Nature..

[33] The Cancer Cell Line Encyclopedia Consortium, Consortium TG of DS in C (2015). Pharmacogenomic agreement between two cancer cell line data sets. Nature..

[34] Bairoch A (2018). The Cellosaurus, a cell-line knowledge resource. J Biomol Tech.

[35] Finn RS, Dering J, Conklin D, Kalous O, Cohen DJ, Desai AJ (2009). PD 0332991, a selective cyclin D kinase 4/6 inhibitor, preferentially inhibits proliferation of luminal estrogen receptor-positive human breast cancer cell lines in vitro. Breast Cancer Res..

[36] Sonnenberg A, Daams H, Van der Valk MA, Hilkens J, Hilgers J (1986). Development of mouse mammary gland: identification of stages in differentiation of luminal and myoepithelial cells using monoclonal antibodies and polyvalent antiserum against keratin. J Histochem Cytochem.

[37] Finn RS, Dering J, Ginther C, Wilson CA, Glaspy P, Tchekmedyian N (2007). Dasatinib, an orally active small molecule inhibitor of both the src and abl kinases, selectively inhibits growth of basal-type/“triple-negative” breast cancer cell lines growing in vitro. Breast Cancer Res Treat.

[38] Guy CT, Webster MA, Schaller M, Parsons TJ, Cardiff RD, Muller WJ (1992). Expression of the neu protooncogene in the mammary epithelium of transgenic mice induces metastatic disease. Proc Natl Acad Sci U S A.

[39] Tchafa AM, Shah AD, Wang S, Duong MT, Shieh AC. Three-dimensional cell culture model for measuring the effects of interstitial fluid flow on tumor cell invasion. J Vis Exp. 2012;(65):4159. 10.3791/4159. 10.3791/4159 PMC3476398 22872144

[40] Sachs N, Secades P, van Hulst L, Kreft M, Song J-Y, Sonnenberg A (2012). Loss of integrin α3 prevents skin tumor formation by promoting epidermal turnover and depletion of slow-cycling cells. Proc Natl Acad Sci U S A.

[41] Ahmed N, Riley C, Rice G, Quinn M (2005). Role of integrin receptors for fibronectin, collagen and laminin in the regulation of ovarian carcinoma functions in response to a matrix microenvironment. Clin Exp Metastasis..

[42] Shirakihara T, Kawasaki T, Fukagawa A, Semba K, Sakai R, Miyazono K (2013). Identification of integrin α3 as a molecular marker of cells undergoing epithelial-mesenchymal transition and of cancer cells with aggressive phenotypes. Cancer Sci.

[43] Gonzales M, Haan K, Baker SE, Fitchmun M, Todorov I, Weitzman S (1999). A cell signal pathway involving laminin-5, alpha3beta1 integrin, and mitogen-activated protein kinase can regulate epithelial cell proliferation. Mol Biol Cell.

[44] Novitskaya V, Romanska H, Kordek R, Potemski P, Kusińska R, Parsons M (2014). Integrin α3β1-CD151 complex regulates dimerization of ErbB2 via RhoA. Oncogene..

[45] Baselga J, Swain SM (2009). Novel anticancer targets: revisiting ERBB2 and discovering ERBB3. Nat Rev Cancer.

[46] Worzfeld T, Swiercz JM, Looso M, Straub BK, Sivaraj KK, Offermanns S (2012). ErbB-2 signals through Plexin-B1 to promote breast cancer metastasis. J Clin Invest.

[47] Varzavand A, Drake JM, Svensson RU, Herndon ME, Zhou B, Henry MD (2013). Integrin α3β1 regulates tumor cell responses to stromal cells and can function to suppress prostate cancer metastatic colonization. Clin Exp Metastasis.

[48] Chary SR, Jain RK (1989). Direct measurement of interstitial convection and diffusion of albumin in normal and neoplastic tissues by fluorescence photobleaching. Proc Natl Acad Sci U S A.

[49] Nguyen-Ngoc K-V, Cheung KJ, Brenot A, Shamir ER, Gray RS, Hines WC (2012). ECM microenvironment regulates collective migration and local dissemination in normal and malignant mammary epithelium. Proc Natl Acad Sci U S A.

[50] Egeblad M, Rasch MG, Weaver VM (2010). Dynamic interplay between the collagen scaffold and tumor evolution. Curr Opin Cell Biol.

[51] Provenzano PP, Eliceiri KW, Campbell JM, Inman DR, White JG, Keely PJ (2006). Collagen reorganization at the tumor-stromal interface facilitates local invasion. BMC Med.

[52] Ramirez NE, Zhang Z, Madamanchi A, Boyd KL, O’Rear LD, Nashabi A (2011). The α_2_β_1_ integrin is a metastasis suppressor in mouse models and human cancer. J Clin Invest.

[53] Hodivala-Dilke KM, DiPersio CM, Kreidberg JA, Hynes RO (1998). Novel roles for alpha3beta1 integrin as a regulator of cytoskeletal assembly and as a trans-dominant inhibitor of integrin receptor function in mouse keratinocytes. J Cell Biol.

[54] Liu S, Yamashita H, Weidow B, Weaver AM, Quaranta V (2010). Laminin-332-beta1 integrin interactions negatively regulate invadopodia. J Cell Physiol.

[55] Ginsberg MH, Partridge A, Shattil SJ (2005). Integrin regulation. Curr Opin Cell Biol.

[56] Ahmadzadeh H, Webster MR, Behera R, Jimenez Valencia AM, Wirtz D, Weeraratna AT (2017). Modeling the two-way feedback between contractility and matrix realignment reveals a nonlinear mode of cancer cell invasion. Proc Natl Acad Sci U S A.

[57] He X, Lee B, Jiang Y (2016). Cell-ECM interactions in tumor invasion. Adv Exp Med Biol.

[58] Kraning-Rush CM, Califano JP, Reinhart-King CA (2012). Cellular traction stresses increase with increasing metastatic potential. PLoS One.

[59] Staunton JR, Doss BL, Lindsay S, Ros R (2016). Correlating confocal microscopy and atomic force indentation reveals metastatic cancer cells stiffen during invasion into collagen I matrices. Sci Rep.

[60] Alluri P, Newman L (2014). Basal-like and triple negative breast cancers: searching for positives among many negatives. Surg Oncol Clin N Am.

