# Supplementary figures and images for: Absence of integrin α3β1 promotes the progression of HER2-driven breast cancer in vivo
Source: Breast Cancer Res. 2019 May 17;21:63. doi: 10.1186/s13058-019-1146-8 (PMC6525362; doi:10.1186/s13058-019-1146-8)

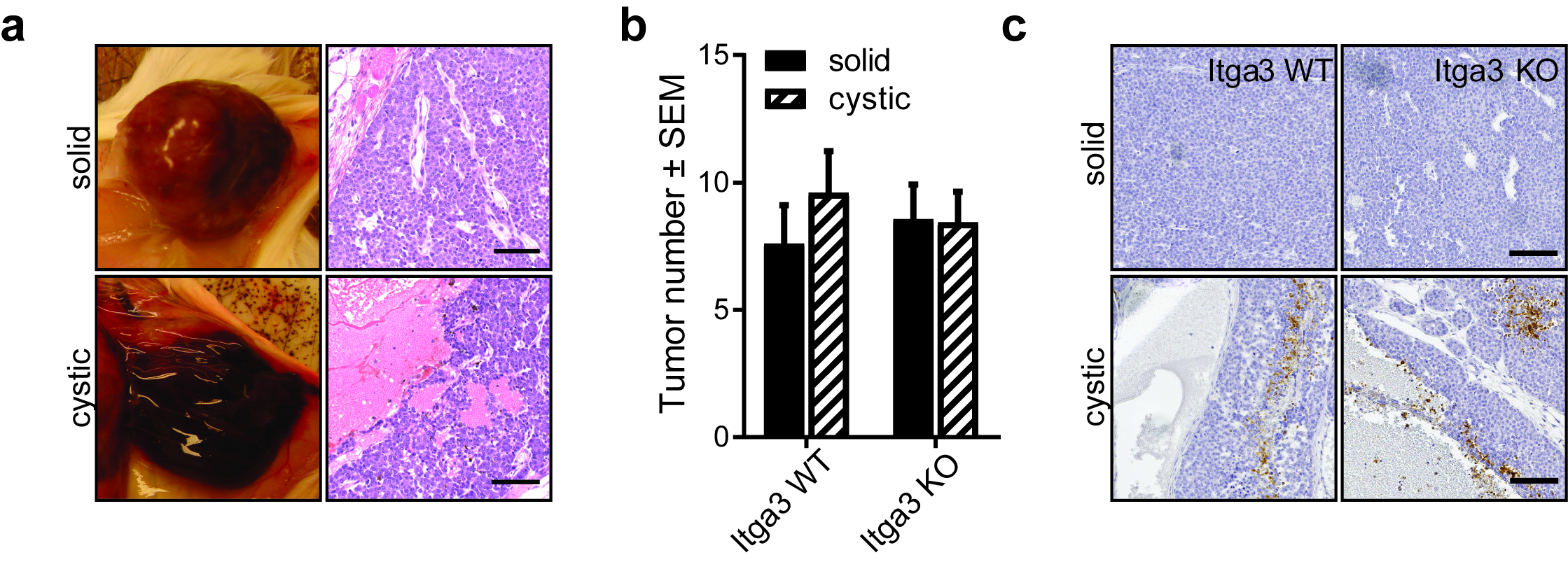

Supplement: Supplementary file 1 — Figure S1. (a) Representative macroscopic and H&E-stained pictures of the two types of tumors detected: solid tumors with high cellularity and cystic tumors which consist of multiple large and small cysts/open areas filled with blood. Scale bar, 100 μm. (b) Quantification of solid vs cystic tumors Itga3 KO and WT mice determined by pathological analysis of excised tumors. No significant differences between both groups could be detected. (c) Immunohistochemical staining for cleaved-Cas3 marker of apoptosis in representative solid (top) and cystic (bottom) tumors of Itga3 KO and WT mice. No positive staining was observed in any of the analyzed solid tumors, and a comparable amount of apoptosis was detected in cystic tumors of Itga3 KO and WT mice. Scale bar, 100 μm. (TIF 4018 kb) [file 13058_2019_1146_MOESM1_ESM.tif]

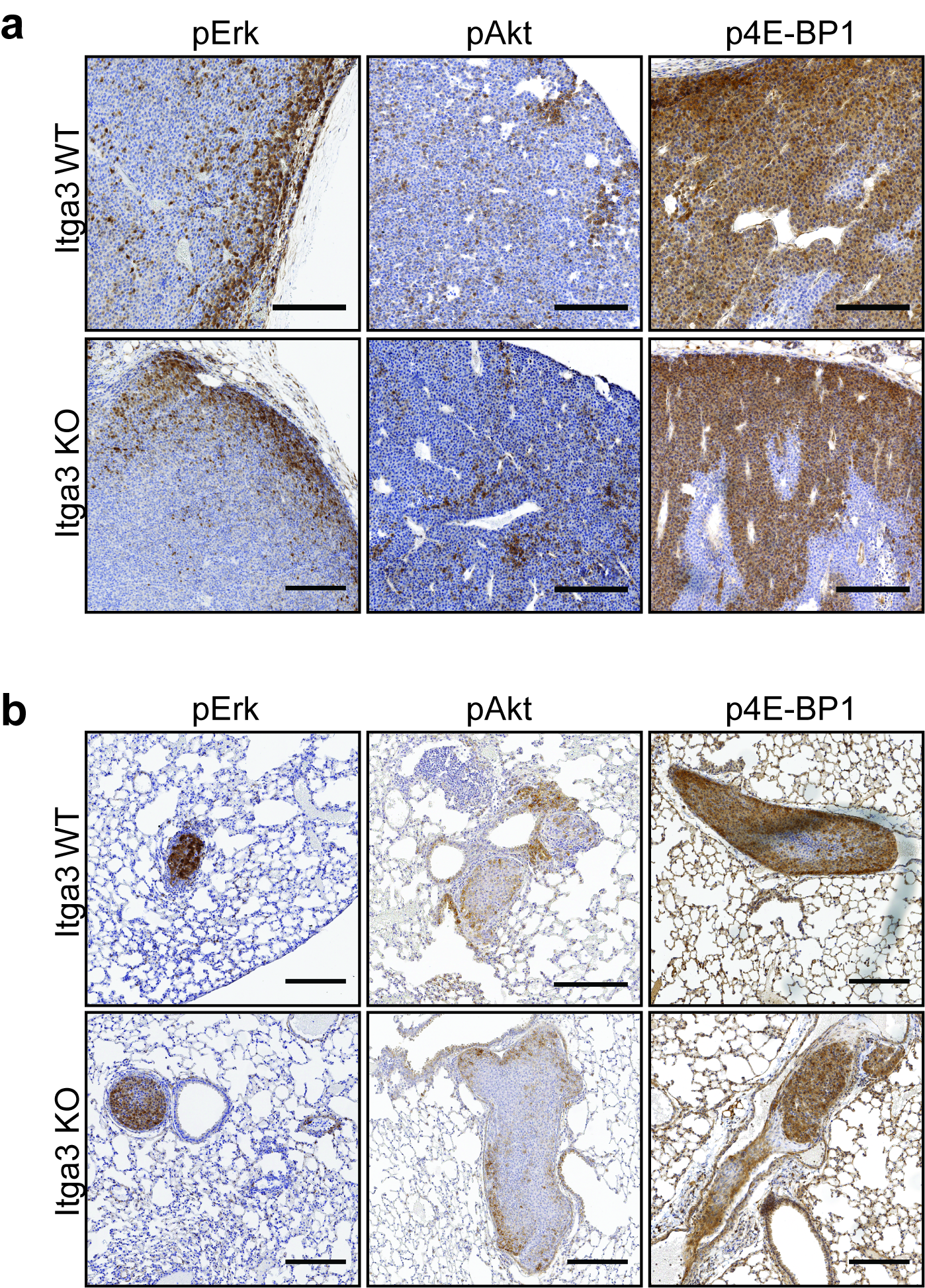

Supplement: Supplementary file 2 — Figure S2. Representative images of immunohistochemical staining of (a) primary tumors and (b) metastases of Itga3 KO and WT mice. No differences were observed in the activation of main HER2-mediated pathways, as seen by pAkt, pErk, and p4E-BP1 staining. Scale bars, 200 μm. (TIF 8790 kb) [file 13058_2019_1146_MOESM2_ESM.tif]

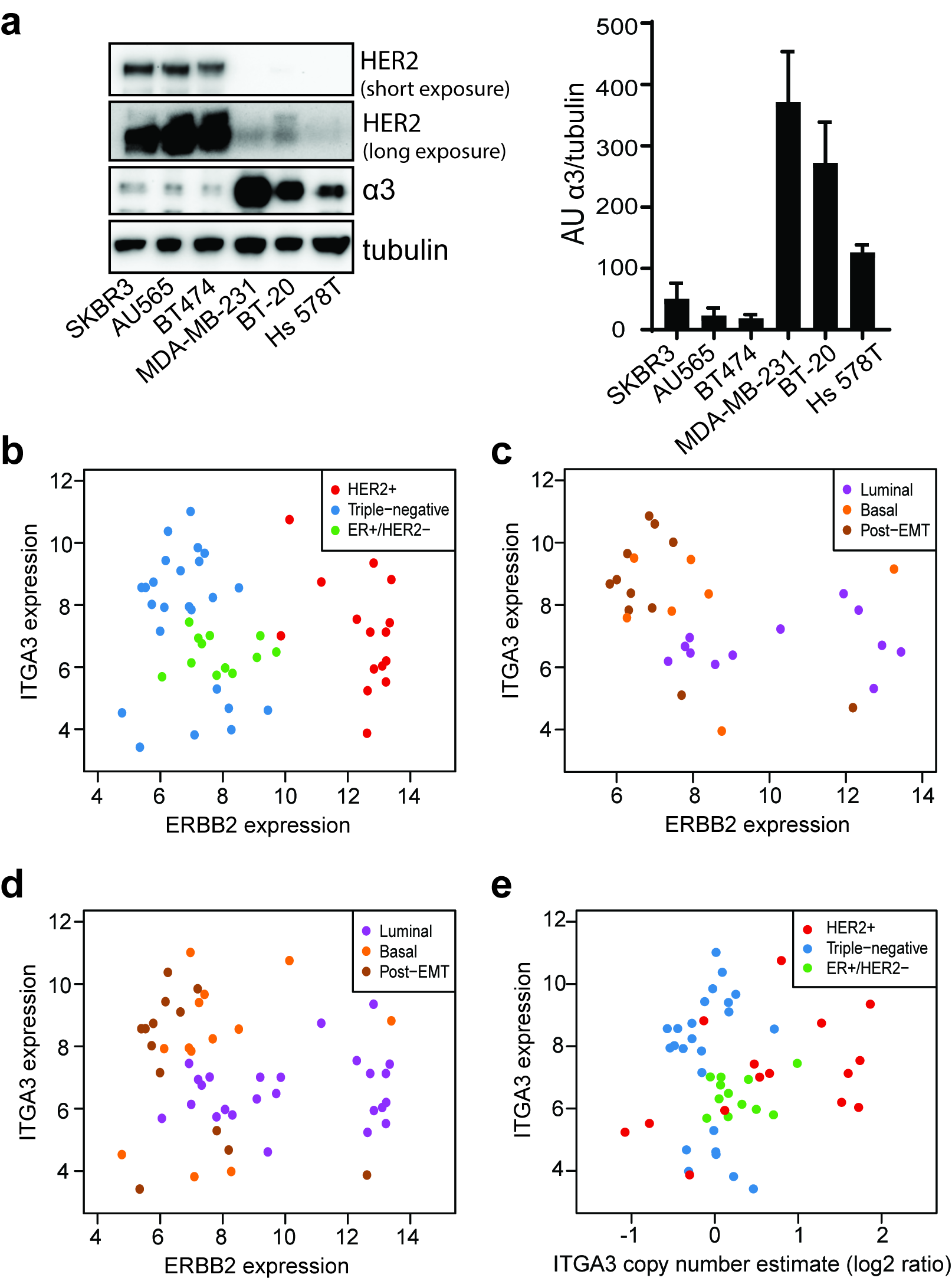

Supplement: Supplementary file 3 — Figure S3. (a) Representative western blot (left) and quantification (right) of three separate experiments of whole cell lysates of triple-negative MDA-MB-231, BT-20, Hs 578T and HER2+ BT474, AU565, and SKBR3 mammary carcinoma cells. HER2-overexpressing cell lines exhibit strongly reduced levels of α3 protein. (b) Scatter plot showing a lack of correlation between ITGA3 and ERBB2 expression in CCLE breast cancer panel (Spearman’s rho − 0.17, P = 0.22, n = 51). (c-d) Scatter plots of ITGA3 and ERBB2 expression of breast cancer cell lines, classified as luminal-, basal-, and post-EMT-like show clustering of luminal-like cell lines to low ITGA3 expression: (c) HER2+ and triple-negative-enriched breast cancer panel [22] (n = 30). (d) CCLE breast cancer cell panel (n = 51). (e) Scatter plot of ITGA3 gene copy number estimates against ITGA3 expression for CCLE dataset. Despite ITGA3 amplification in several HER2+ cell lines, their expression of ITGA3 remains relatively low. (TIF 1468 kb) [file 13058_2019_1146_MOESM3_ESM.tif]

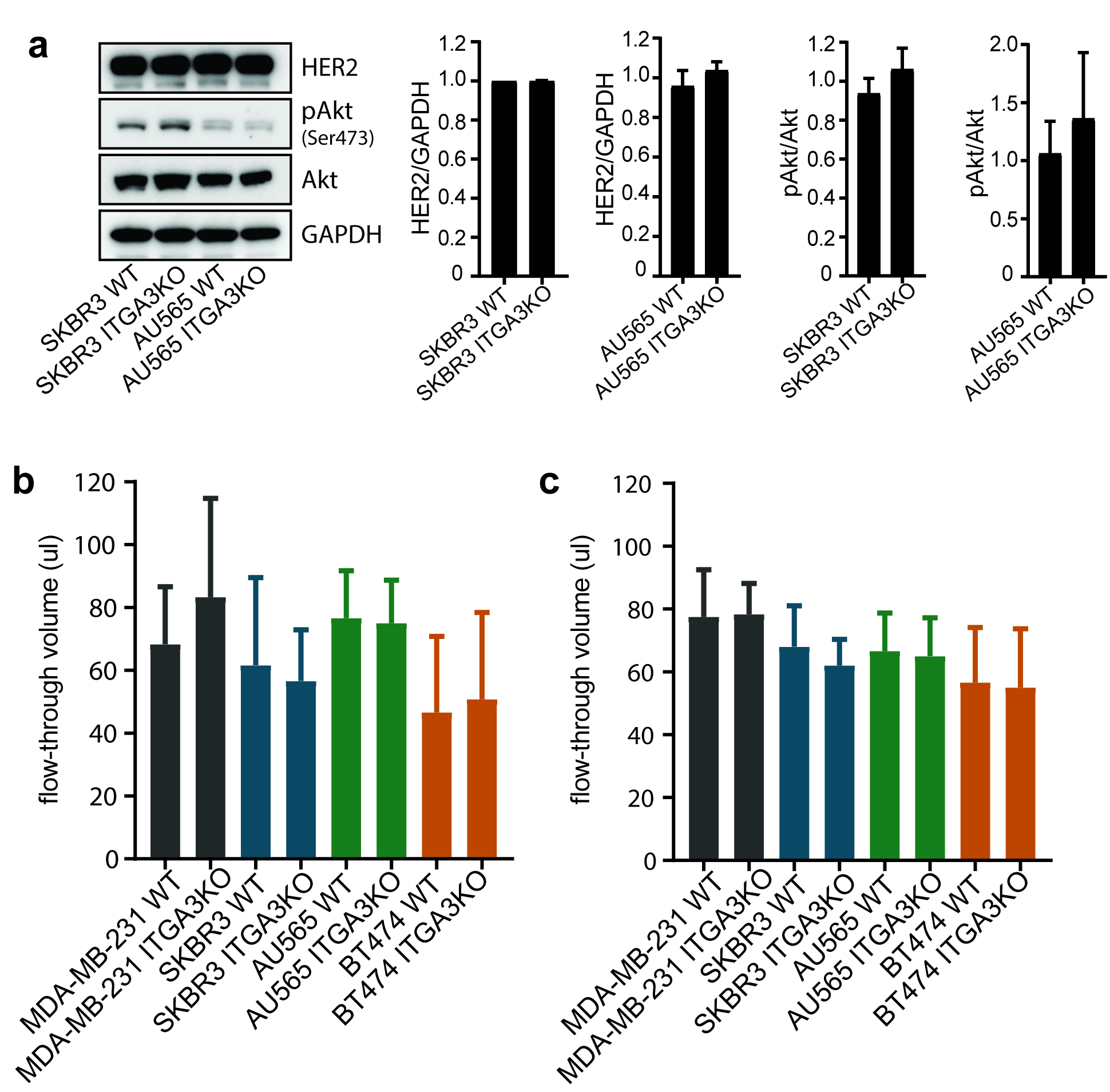

Supplement: Supplementary file 4 — Figure S4. (a) Representative western blot (left) and quantification (right) of three separate experiments of whole cell lysates of WT and ITGA3 KO AU565 and SKBR3 mammary carcinoma cells. No differences in the levels of HER2 or in Akt signaling were observed between ITGA3 KO and WT cells. (b-c) Volume of medium, passing the (b) mixture of collagen I and Matrigel and (c) Matrigel only during the invasion assays under interstitial flow conditions (mean ± SD). No significant differences were observed (one-way ANOVA). (TIF 1500 kb) [file 13058_2019_1146_MOESM4_ESM.tif]

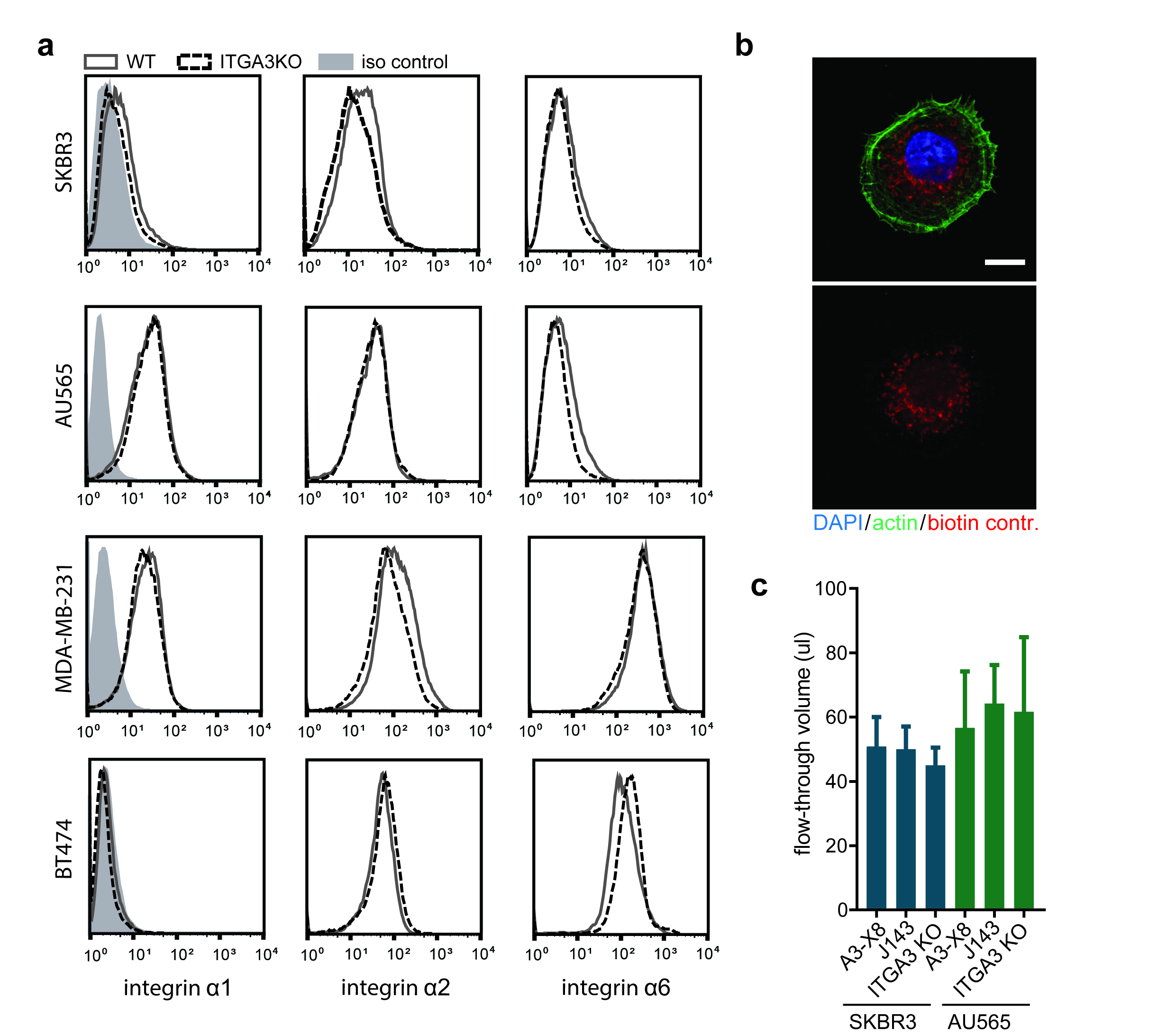

Supplement: Supplementary file 5 — Figure S5. (a) Flow cytometry histograms of surface expression of collagen-binding integrins α1 and α2 and laminin-binding integrin α6 in ITGA3 KO and WT mammary carcinoma cells. (b) Representative image of SKBR3 ITGA3 WT cells, stained with biotin-conjugated secondary antibody as a background control (scale bar, 10 μm). (c) Volume of medium, passing the mixture of collagen I and Matrigel during the invasion assays under interstitial flow conditions and with the addition of α3-function blocking (J143) and control (A3-X8) antibodies (mean ± SD). No significant differences were observed (one-way ANOVA). (TIF 2113 kb) [file 13058_2019_1146_MOESM5_ESM.tif]
